# Comprehensive methylome sequencing reveals prognostic epigenetic biomarkers for prostate cancer mortality

**DOI:** 10.1002/ctm2.1030

**Published:** 2022-09-30

**Authors:** Ruth Pidsley, Dilys Lam, Wenjia Qu, Timothy J. Peters, Phuc‐Loi Luu, Darren Korbie, Clare Stirzaker, Roger J. Daly, Phillip Stricker, James G. Kench, Lisa G. Horvath, Susan J. Clark

**Affiliations:** ^1^ Garvan Institute of Medical Research Sydney New South Wales Australia; ^2^ School of Clinical Medicine St Vincent's Healthcare Clinical Campus, Faculty of Medicine and Health, UNSW Sydney Sydney New South Wales Australia; ^3^ Centre for Personalised Nanomedicine Australian Institute for Bioengineering and Nanotechnology The University of Queensland St. Lucia Queensland Australia; ^4^ Cancer Research Program and Department of Biochemistry and Molecular Biology Biomedicine Discovery Institute Monash University Clayton Victoria Australia; ^5^ Department of Urology St. Vincent's Prostate Cancer Centre Sydney New South Wales Australia; ^6^ Department of Tissue Pathology NSW Health Pathology Royal Prince Alfred Hospital Camperdown Sydney New South Wales Australia; ^7^ Chris O'Brien Lifehouse, Camperdown Sydney New South Wales Australia; ^8^ University of Sydney Sydney New South Wales Australia; ^9^ Present address: School of Molecular Sciences, The University of Western Australia, Crawley, Western Australia 6009, Australia; ^10^ Present address: Harry Perkins Institute of Medical Research, Nedlands, Western Australia 6009, Australia

**Keywords:** biomarkers, *CACNA2D4*, DNA methylation, prognosis, prostate cancer, survival

## Abstract

**Background:**

Prostate cancer is a clinically heterogeneous disease with a subset of patients rapidly progressing to lethal‐metastatic prostate cancer. Current clinicopathological measures are imperfect predictors of disease progression. Epigenetic changes are amongst the earliest molecular changes in tumourigenesis. To find new prognostic biomarkers to enable earlier intervention and improved outcomes, we performed methylome sequencing of DNA from patients with localised prostate cancer and long‐term clinical follow‐up.

**Methods:**

We used whole‐genome bisulphite sequencing (WGBS) to comprehensively map and compare DNA methylation of radical prostatectomy tissue between patients with lethal disease (*n* = 7) and non‐lethal (*n* = 8) disease (median follow‐up 19.5 years). Validation of differentially methylated regions (DMRs) was performed in an independent cohort (*n* = 185, median follow‐up 15 years) using targeted multiplex bisulphite sequencing of candidate regions. Survival was assessed via univariable and multivariable analyses including clinicopathological measures (log‐rank and Cox regression models).

**Results:**

WGBS data analysis identified cancer‐specific methylation patterns including CpG island hypermethylation, and hypomethylation of repetitive elements, with increasing disease risk. We identified 1420 DMRs associated with prostate cancer‐specific mortality (PCSM), which showed enrichment for gene sets downregulated in prostate cancer and *de novo* methylated in cancer. Through comparison with public prostate cancer datasets, we refined the DMRs to develop an 18‐gene prognostic panel. Applying this panel to an independent cohort, we found significant associations between PCSM and hypermethylation at *EPHB3*, *PARP6*, *TBX1*, *MARCH6* and a regulatory element within *CACNA2D4*. Strikingly in a multivariable model, inclusion of *CACNA2D4* methylation was a better predictor of PCSM versus grade alone (Harrell's C‐index: 0.779 vs. 0.684).

**Conclusions:**

Our study provides detailed methylome maps of non‐lethal and lethal prostate cancer and identifies novel genic regions that distinguish these patient groups. Inclusion of our DNA methylation biomarkers with existing clinicopathological measures improves prognostic models of prostate cancer mortality, and holds promise for clinical application.

## Graphical abstract

Comprehensive whole‐genome bisulphite sequencing of prostate cancer tissue identified 1420 differentially methylated regions (DMRs) between patients with lethal disease versus non‐lethal disease. DMRs were used to develop a targeted sequencing biomarker panel which validated cancer‐specific methylation patterns in an independent cohort (*n* = 185). The epigenetic biomarker panel improves accuracy of identifying patients at risk of death compared to existing clinicopathological markers.

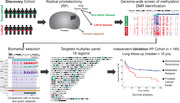

## 1 BACKGROUND

Prostate cancer is the second‐most common cancer diagnosed in men. Currently, 80% of patients present with localised disease^1^ and are typically recommended for active surveillance or radical prostatectomy (RP) surgery. However, of the patients treated with RP, up to 40% experience a biochemical recurrence (BCR), with 5%–10% of patients progressing to lethal‐metastatic prostate cancer.^2^ Current prognostic clinicopathological factors, including prostate‐specific antigen (PSA) levels, ISUP Grade Group pathological score, pathological T‐category and surgical margin status, lack sensitivity and specificity in predicting progression or outcome, particularly in patients with localised intermediate risk prostate cancer.^3^ There is a critical need for more reliable and accurate prognostic biomarkers to guide more personalised management.

Molecular profiling promises novel prognostic biomarkers. DNA methylation at cytosine‐guanine (CpG) sites is of particular interest as it is one of the earliest molecular changes to occur in prostate cancer and is preserved through metastatic progression.^4^ Early methylation studies, which focused on candidate gene promoters, revealed a small number of methylation alterations associated with prognosis.^5^ Recent studies have widened the genomic ‘search area’ by conducting unbiased epigenome‐wide microarray‐based techniques to identify novel prognostic markers.^6^ However, microarray‐based technologies only cover a small percentage (∼1%–3%) of the ∼28 million CpG sites in the genome.^7^ Moreover, most studies have focused on prognostic methylation markers of BCR, but BCR is not always a suitable surrogate for prostate cancer‐specific mortality (PCSM).^8^ Long‐term follow‐up (>15 years) is needed for the clinically important endpoints of metastatic relapse (MR) and PCSM to manifest.^9^

In this study, we applied single‐base resolution methylation sequencing technologies to two independent prostate cancer cohorts with long‐term follow‐up clinical data (median: >15 years). We profiled DNA from primary prostate cancer tissue obtained at RP in a discovery cohort using whole‐genome bisulphite sequencing (WGBS), which can measure methylation at almost every CpG in the genome. We identified a set of differentially methylated regions (DMRs) between patients who died ≤10 years post‐RP and those still alive >10 years post‐RP. Multiplex bisulphite PCR sequencing (MBPS) was then used to validate a subset of these DMRs in RP tissue from a validation cohort^8^, ^10^ using PCSM, MR and BCR as survival endpoints.

## 2 METHODS

### 2.1 Study populations

#### 2.1.1 Discovery cohort

The discovery cohort consists of 15 patients that underwent RP to treat clinically localised prostate cancer between the years 1989 and 2003, at the St. Vincent's Hospital, Sydney, Australia. Seven patients died of prostate cancer within 10 years of RP (‘lethal’ group), whilst the remaining eight patients were alive at last follow‐up (minimum: 13.5 years, median: 19.5 years) (‘non‐lethal’ group). The non‐lethal group was matched to the lethal group according to tumour grade, clinical stage and local invasion (seminal vesicle invasion and extracapsular). As additional controls, adjacent normal tissue from four prostate cancer patients was included.

#### 2.1.2 Validation cohort

An independent group of 186 prostate cancer patients was used for validation of the prognostic significance of 18 DMRs identified in the discovery cohort. This group of patients, selected with ISUP Grade Group 2 or higher, is a subset of patients that underwent RP treatment between 1997 and 2003 at the St. Vincent's Hospital.^8^, ^10^ Over an extensive follow‐up period (median: 15 years, range: 0.8–22 years), 86 patients experienced BCR, 25 patients had MR and 16 patients died of prostate cancer.

### 2.2 Tumour tissue preparation, DNA extraction and bisulphite conversion

Archival formalin‐fixed paraffin‐embedded (FFPE) prostate tumour tissue blocks were obtained from RP specimens for both cohorts. Haematoxylin and eosin staining on prostate tissue specimens was reviewed by (uro)pathologists to mark and confirm the presence and location of prostate cancer tumour areas. For each patient, five 1 mm tumour tissue cores from within histologically verified tumour region (at least 50% neoplastic cells (typically >70%)) were taken for genomic DNA extraction. For the four normal adjacent samples, five 1 mm tissue cores were taken per patient from regions with 0% neoplastic cells (no carcinoma, intraductal carcinoma of prostate, prostatic intraepithelial neoplasia, proliferative inflammatory atrophy, or necrosis). DNA was extracted using the AllPrep FFPE DNA/RNA Kit (Qiagen, Cat. No. 80234), quantified with the Qubit dsDNA HS Assay Kit (Life Technologies, USA), and stored at ‐80°C until use. Extracted DNA was bisulphite treated using the EZ DNA Methylation‐Lightning Kit (Zymo Research, USA, Cat. Nos. D5030 and D5033) according to the manufacturer's instructions.

### 2.3 DNA methylation profiling (discovery)

#### 2.3.1 WGBS library preparation, quality control and sequencing

WGBS libraries were prepared using the EpiGnome Methyl‐Seq Kit (Epicentre, EGMK81312), according to the manufacturer's protocol. Library quality was assessed with the Agilent 2100 Bioanalyzer using the High‐Sensitivity DNA Kit (Agilent, CA, USA). DNA was quantified using the KAPA Library Quantification Kit by quantitative PCR (KAPA 6 Biosystems). Libraries from patient specimens were analysed with 70 bp paired‐end sequencing on the Illumina HiSeq 2500 platform using TruSeq Rapid SBS Kit ‐ HS (50 cycle) and TruSeq Rapid PE Cluster Kit ‐ HS. Six samples were multiplexed across two lanes. Sequencing was performed multiple times to gain sufficient coverage.

#### 2.3.2 WGBS data processing and statistical analysis

Adaptor sequences and poor quality bases were removed using Trim Galore (version 0.2.8, http://www.bioinformatics.babraham.ac.uk/projects/trim_galore/) in paired‐end mode with default parameters. bwa‐meth (version 0.10)^11^ was used to align reads to hg19 using default parameters. PCR duplicates were removed using Picard (version 1.91, http://broadinstitute.github.io/picard). Count tables of the number of methylated and unmethylated bases sequenced at each CpG site in the genome were constructed using the ‘tabulate’ module of bwa‐meth and BisSNP (version 0.82.2)^12^ with default parameters. The processed WGBS data were exported and uploaded to NCBI GEO repository GSE158927 (https://www.ncbi.nlm.nih.gov/geo) as ‘GSE158927_BigTable.tsv.gz’; a tsv file providing coverage and methylation data for each sample (columns) at each CpG site (rows). In the column names, chr: chromosome, position: genomic position; then for each sample, C: count methylated and cov: total coverage.

All statistical analyses were conducted using R (version ≥3.2.2)^13^ and all scripts uploaded to public Github repository (https://github/clark‐lab/ProstateLethal). For dimensionality‐reduction analysis, principal components analysis (PCA) and plots were generated using the vegan package in R.^14^ Plots of average methylation by CpG context were created using the *methWindowRatios* and *methDensityPlot* functions implemented in the R package aaRon (https://github.com/astatham/aaRon). For this analysis, a bed‐formatted annotation file of CpG islands was downloaded from UCSC hg19 using the rtracklayer Bioconductor package.^15^ CpG shores were defined as the regions 2000 bp either side of each CpG island, and all genomic regions >2000 bp distant were defined as non‐CpG. Annotation data for repetitive elements (LINE‐1, LTR and Alu) corresponding to the RepeatMasker database on USCS were downloaded using the REMP package (version 1.10.1).^16^

DMRs between lethal and non‐lethal patients were called using DMRcate^17^ with parameters C = 50, min.cpgs = 5 and a Stouffer threshold of <0.05, with DSS^18^ used as an initial differentially methylated loci caller. Methylation values of samples at all DMRs were visualised as a heatmap with dendrogram, using the *heatmap.2* function in the gplots R package.^19^ Individual DMRs were visualised using the *DMR.plot* function in DMRcate.^17^ For downstream analysis, DMRs were separated according to whether they were hyper‐ or hypomethylated in lethal patients. DMRs were annotated for overlap and proximity with genetic features using the *annotateRegions* function implemented in the R package aaRon. For investigation of the lethal DMRs in lymph node carcinoma of the prostate (LNCaP) cells and prostate epithelial cells (PrEC), we used in‐house chromatin state and WGBS data that was generated and processed as previously described.^7^, ^20^ The *import.bw* function in the rtracklayer Bioconductor package^15^ was used to extract LNCaP and PrEC WGBS methylation data, from bigwig files, at the DMR locations.

For functional enrichment analysis, DMRs were flagged for overlaps with any GeneHancer Double Elite^21^ region. The list of interacting genes for DMR‐overlapping enhancers and/or promoters was then tested for gene set enrichment in gene sets from the Molecular Signatures Database (MSigDB) version 6.1^22^ using the RITAN and RITANdata Bioconductor packages.^23^ The background was defined as the complete list of GeneHancer Double Elite genes with known interactions and enhancer and/or promoter regions, and terms with a false discovery rate (FDR) <0.05 were called as significant. The results were filtered to only include gene sets with between 15 and 500 genes, and visualised by adapting functions from the enrichplot Bioconductor package (https://github.com/GuangchuangYu/enrichplot).

### 2.4 Validation of candidate DMRs in public datasets

From the top list of DMRs, we identified 18 to be developed into a diagnostic panel, by identifying DMRs with a large mean difference between ‘lethal’ and ‘non‐lethal’ RP samples (≥30%) and confirming their suitability via comparison with public datasets.

#### 2.4.1 Processing and analysis of public methylation and expression data from prostate tissue

Prostate adenocarcinoma (PRAD) HumanMethylation450 BeadChip (HM450K) methylation data were downloaded from The Cancer Genome Atlas (TCGA) Data Portal website (http://tcga‐data.nci.nih.gov/tcgafiles) and processed as described in Pidsley et al.,^24^ giving 414 133 CpG sites from 437 samples (*n* = 45 normal and *n* = 392 tumour). We identified 96 CpG probes that overlapped with 17 out of 18 of the WGBS candidate DMRs. We then calculated the average methylation of all CpGs within a DMR, to obtain a methylation value for each of the 17 DMRs for each sample.

PRAD processed RNA‐seq V2 data (level 3) was downloaded from the TCGA Data Portal website. We used R to perform Pearson's correlation tests between patient‐matched DMR methylation and the RNA‐seq gene expression of the nearest‐protein coding gene.

#### 2.4.2 Processing and analysis of public methylation data from whole blood

Processed HM450K methylation data from human whole blood samples were downloaded from the NCBI GEO database (https://www.ncbi.nlm.nih.gov/geo/) with accession GSE40279^25^ and imported into the R environment^13^ using the *minfi* Bioconductor package.^26^ We subset the methylation *β* values to only include the blood methylation data for male samples, leaving *n* = 318 samples for analysis. We identified 91 CpG probes that overlapped with 16 out of 18 of the WGBS candidate DMRs (note: chromosome X data were not available for this dataset). We then calculated the average methylation of all CpGs within a DMR, to obtain a methylation value for each of the 16 DMRs for each sample.

#### 2.4.3 Analysis of public methylation and chromatin state data from cell lines

Further investigation of the potential functional importance of the 18 candidate DMRs was performed using the LNCaP and PrEC WGBS methylation and chromatin state data (also used to investigate the full set of DMRs above). bedGraph files were generated to visualise all the public validation data in the IGV genome browser.^27^

### 2.5 Multiplex bisulphite PCR sequencing (validation)

#### 2.5.1 MBPS panel design, optimisation and sequencing

A panel of 18 candidate prognostic DMRs was developed into an MBPS assay, following the protocol detailed in a study by Lam et al. ^28^ Briefly, MBPS primers targeting the 18 selected DMR regions were designed using the custom multiplex‐specific primer design software, PrimerSuite (www.primer‐suite.com),^29^, ^30^ with the following parameters: 105–150 bp amplicon size, oligo melting temperature of 54°C, sodium concentration of 50 mM and maximum of one CpG dinucleotide allowed within primers. The 18 designed primers were pooled together, and the optimal annealing temperature and primer concentration were determined to be 56°C and 10 μM, respectively, and 28 PCR cycles. Primer sequences, location and the number of CpG dinucleotides interrogated are given in Table S1 and amplicon locations relative to WGBS DMR are visualised in Figure S1.

The optimised PCR conditions were used to run the MBPS assay on *n* = 186 samples from the validation cohort. PCR amplification of the 18 primers was performed on bisulphite‐treated patient DNA (∼6 ng) in triplicate, with patients randomly distributed across two 384‐well plates. Individual libraries (per patient) were pooled at equal amounts (96 samples per sequencing run), and diluted to 10 nM following library quantification, ready for sequencing. Sequencing was performed on the Illumina NextSeq sequencer (Illumina, CA, USA), with sample preparation for sequencing performed according to Illumina's instructions (1.8 pM, 20% PhiX Control v3 [Illumina, FC‐110‐3001]). A series of methylated‐control DNA samples with known methylation percentages (0%, 1%, 25%, 50%, 75%, 100%) were also sequenced, prepared by proportionally mixing 0% and 100% methylated DNA (whole‐genome‐amplified non‐methylated and methylated DNA, Cat. No. D5013). These methylated‐control DNA samples were used to assess PCR bias of each region examined.^31^

#### 2.5.2 MBPS data processing and quality control

We used the *MethPanel* workflow^31^ to preprocess and align MBPS reads to predefined regions (based on PrimerSuite software output, Table S1) of the reference genome hg19 build. Specifically, FASTQ files were trimmed to produce high‐quality reads with base quality ≥30, read length ≥20 bp and to clip 1 bp from both reads (https://github.com/FelixKrueger/TrimGalore). Bismark (version 0.22.3)^32^ was used to map these trimmed reads to the predefined reference genome, allowing one non‐bisulphite mismatch per read, with all other parameters kept to their default values.

For each bam file produced by Bismark, MethPanel was used to perform calculation of DNA methylation levels and merge all samples into a single table. Further quality control was performed to remove amplicons and samples with <100× coverage from the methylation table. PCR bias was assessed using methylation‐control samples, as described in Lam et al.^28^ This showed minimal bias across the amplicons, so bias‐correction was not applied to the methylation data prior to prognostic analysis. Methylation values were then averaged across the CpGs within each region.

#### 2.5.3 Survival analysis

The prognostic value of the candidate DMRs was tested in an independent cohort of 186 patients for validation, using survival outcomes: BCR, defined as a serum PSA concentration ≥0.2 ng/ml increasing over a 3‐month period; MR, determined by biopsy or positive scan(s) confirming local, visceral or bony metastasis; and PCSM, with deaths identified from the NSW State Cancer Registry and cause of death confirmed by contacting patients’ general practitioner and through review of medical records. For survival analysis, Kaplan–Meier, log‐rank tests and univariable and multivariable Cox proportional hazard models were performed, with significance set at *p* <.05. Known prognostic clinicopathological factors were assessed as dichotomous variables: ISUP Grade Groups (2 vs. ≥3), pathological T‐category (≤pT2 vs. ≥pT3), pre‐operative PSA levels (<10 ng/ml vs. ≥10 ng/ml) and surgical margin status (negative vs. positive). One of the patients was found to have missing pre‐operative PSA data, so was excluded from survival analysis leaving *n* = 185 patients. For analyses using methylation markers, patients were dichotomised into high/low methylation groups for each of the 17 DMRs surviving quality control, based on the methylation value at the 75th percentile cut‐off. For multivariable Cox analyses, the Statistically Equivalent Signatures (SES) feature selection algorithm, part of the ‘MXM’ R package, was used to identify minimal‐size predictive signatures with maximal predictive power by performing a variant of forward selection.^33^ Using the inbuilt conditional independence test for survival analysis (Cox regression, ‘censIndCR’ test), the input variables were ISUP Grade Group, pathological T‐category, PSA level, surgical margin status and methylation at our 17 DMRs. Harrell's concordance index was used to measure the predictive discrimination of the multivariable Cox proportional hazard models for the three survival outcomes, and to assess the additive prognostic effect of methylation markers on existing clinicopathological markers.^34^ This was further investigated through a time‐dependent receiver operating characteristic (ROC) analysis of the multivariable Cox regression models using the ‘risksetROC’ package in R,^35^ with *CACNA2D4* methylation included and excluded from the final model. Area under the curves (AUC) were calculated for survival at 1, 5, 10 and 15 years post‐RP.

To ensure that between‐patient variability in the sequencing coverage of the *CACNA2D4* DMR was not affecting the results, original bam files were downsampled using SAMtools (version 1.12)^36^ to 1000, 10 000, 50 000 and 100 000 reads (or maximum number of reads for each patient if lower). Downsampled datasets were processed and analysed as described above to generate results for log‐rank tests, univariable and multivariable Cox proportional hazard models.

#### 2.5.4 Characterisation of *CACNA2D4*

The GeneHancer list of Double Elite^21^ regions was used to identify overlap between the *CACNA2D4* DMR and known enhancer/promoter regions and their putative target genes. To determine whether *CACNA2D4* expression changes were observable in patient specimens, we used the TCGA PRAD processed RNA‐seq V2 data (described above) and performed Student's *t*‐test to test for differential expression between tumour and normal specimens.

## 3 RESULTS

### 3.1 Methylome profiling of primary prostate cancers reveals global and site‐specific methylation differences between men with non‐lethal and lethal disease

To identify DNA methylation changes associated with prostate cancer survival, we first curated an archival cohort of 15 patients with localised prostate cancer, who had undergone RP, with *n* = 8 patients with non‐lethal disease (alive >10 years post‐RP) and *n* = 7 patients with lethal disease (dead of prostate cancer ≤10 years post‐RP). Groups were well‐matched for the clinical characteristics that are typically used for disease prognosis (Table 1A).

**TABLE 1:** Clinicopathological characteristics: (A) discovery and (B) validation cohorts

(A) Discovery cohort		
Characteristic	Non‐lethal	Lethal
Number of patients	8	7
Age at RP, mean ± SD (range)	60.3 ± 2.1 (57–63)	62.7 ± 5.5 (54–72)
ISUP Grade Groups		
1 (Gleason score ≤6), *n* (%)	1 (12.5)	1 (14.3)
2 (Gleason score 3 + 4), *n* (%)	4 (50.0)	1 (14.3)
3 (Gleason score 4 + 3), *n* (%)	1 (12.5)	3 (42.8)
4 (Gleason score 8), *n* (%)	2 (25.0)	2 (28.6)
Pre‐operative PSA (ng/ml), mean ± SD (range)	11.58 ± 4.4 (5.8–18.4)	18.8 ± 16.9 (2.0–32.4)
Pathological T‐category		
pT2, *n* (%)	1 (12.5)	1 (14.3)
pT3, *n* (%)	7 (87.5)	6 (85.7)
Positive margin status	6 (75.0)	4 (57.2)
Follow‐up (years), median (range)	19.5 (13.5–24.8)	6.6 (2.3–10)
*Clinical outcome*		
Biochemical recurrence, *n* (%)	4 (50.0)	7 (100.0)
Metastatic relapse, *n* (%)	0 (0.0)	6 (85.7)^a^

(B) Validation cohort		
Characteristic		
Number of patients	185	
Age at RP, mean ± SD (range)	61.9 ± 5.8 (46–73)	
ISUP Grade Groups		
2 (Gleason score 3 + 4), *n* (%)	119 (64.3)	
3 (Gleason score 4 + 3), *n* (%)	34 (18.4)	
4 (Gleason score 8), *n* (%)	19 (10.3)	
5 (Gleason score 9, 10), *n* (%)	13 (7.0)	
Pre‐operative PSA (ng/ml), mean ± SD (range)	10.3 ± 7.3 (1.6–63.0)	
PSA <10 ng/ml, *n* (%)	115 (62.2)	
PSA ≥10 ng/ml, *n* (%)	70 (37.8)	
Pathological T‐category		
≤pT2, *n* (%)	89 (48.1)	
≥pT3, *n* (%)	96 (51.9)	
Surgical margin status^b^		
Negative, *n* (%)	91 (49.2)	
Positive, *n* (%)	93 (50.3)	
Follow‐up (years), median (range)	15 (0.8–22)	
*Clinical outcome*		
Biochemical recurrence, *n* (%)	86 (46.5)	
Metastatic relapse, *n* (%)	25 (13.5)	
Prostate cancer‐specific mortality, *n* (%)	16 (8.6)	

Abbreviations: PSA, prostate‐specific antigen; pT, pathological T‐category; RP, radical prostatectomy; SD, standard deviation.

^a^ Date of metastatic relapse for one patient in the Lethal group not reported.

^b^ Margin status for one patient was not reported.

**FIGURE 1:**
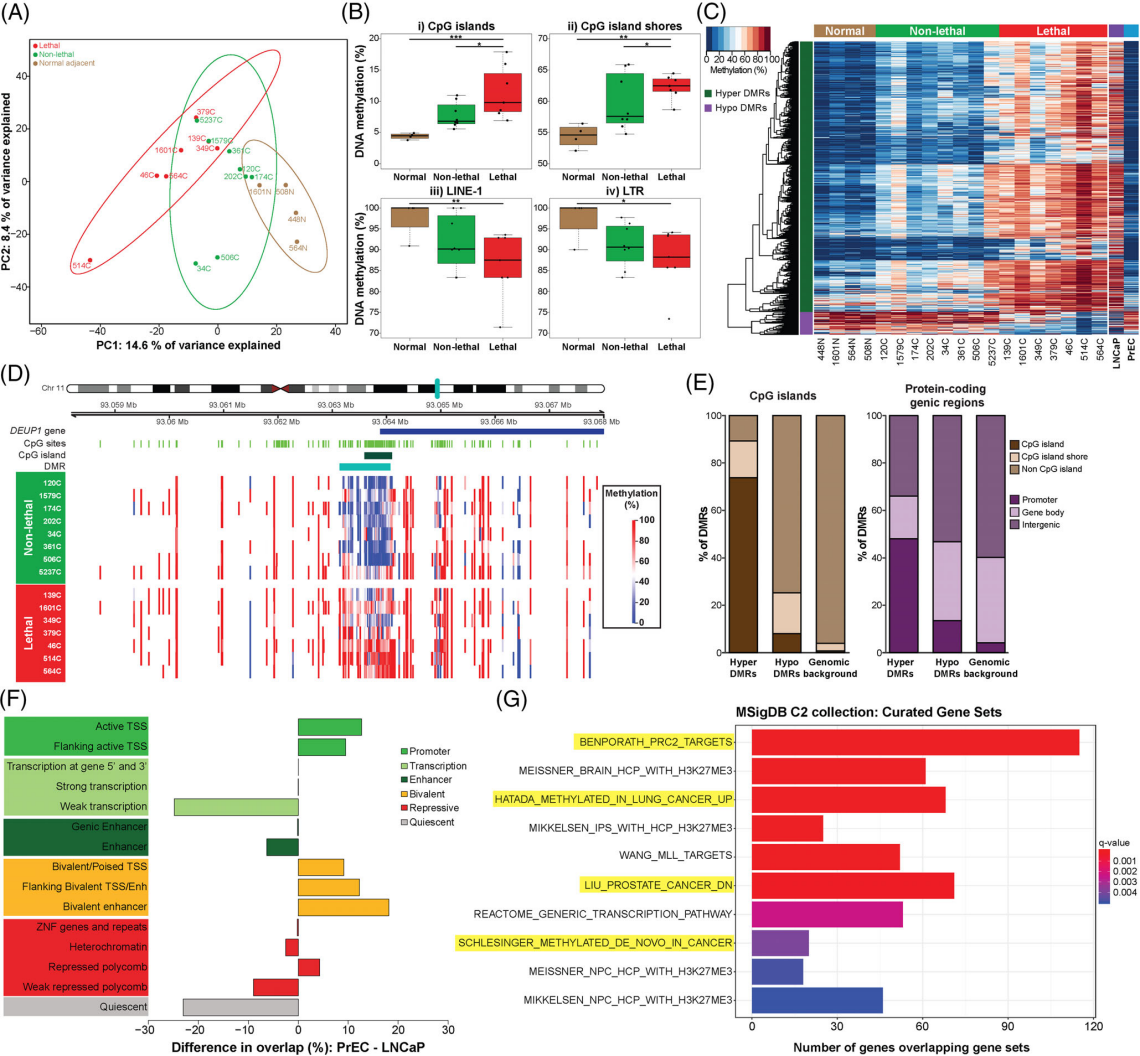
Discovery cohort findings. (A) Principal component analysis of variation in genome‐wide DNA methylation across cancer tissue from patients with lethal (red) and non‐lethal (green) disease, and normal adjacent tissue (light brown). (B) Boxplots of genome‐wide DNA methylation levels in normal adjacent tissue, non‐lethal and lethal patients, split across different CpG contexts: (i) CpG islands, (ii) CpG island shores, (iii) LINE‐1 repetitive elements and (iv) long tandem repeats (LTRs). Each dot indicates the median methylation value for each patient. (C) Heatmap of all 1420 differentially methylated regions (DMRs) (hypermethylated: dark green; hypomethylated: purple), comparing methylation across normal adjacent (brown), non‐lethal (green) and lethal (red) patient samples, alongside lymph node carcinoma of the prostate (LNCaP) (purple) and prostate epithelial cells (PrEC) (blue) prostate cancer cell lines. (D) DMRcate heatmap of a representative DMR (*DEUP1—*DMR #9, turquoise bar) showing methylation across individual patients in the non‐lethal (green) versus lethal (red) groups. The *DEUP1* gene is represented by a dark blue bar, with the promoter CpG island represented by a dark green bar. (E) Bar graphs of the percentage overlap of DMRs with different genomic features. Left panel: CpG island (dark brown), CpG island shore (light brown) and non‐CpG island (brown). Right panel: promoter (dark purple), gene body (light purple) and intergenic regions (purple). (F) Bar graph of the percentage difference in overlap between PrEC and LNCaP ChromHMM states with all hypermethylated DMRs. The putative regulatory elements from ChromHMM segmentation data have been grouped into promoter (active transcription start site (TSS), flanking active TSS) (green), transcription (transcription at 5′ and 3′, strong transcription, weak transcription) (light green), enhancer (genic enhancer, enhancer) (dark green), bivalent (bivalent/poised TSS, flanking bivalent TSS, bivalent enhancer) (light orange), repressive (ZNF genes and repeats, heterochromatin, repressed polycomb, weak repressed polycomb) (red) and quiescent (light grey). (G) Top 10 gene sets enriched in the hypermethylated DMRs, from the Molecular Signatures Database (MSigDB) C2 collection. Significance of enrichment denoted by *q*‐value, and terms highlighted in yellow refer to cancer‐related gene sets.

We performed WGBS on FFPE tissue from RP, and adjacent normal tissue specimens from four patients. Initial visualisation of the data using PCA showed that, at the global level, variation in genome‐wide DNA methylation was able to distinguish between the patient groups (Figure 1A). The first principal component explains 14.6% of the variance in the methylation data and shows a gradation between the normal, non‐lethal and lethal disease groups. Sub‐setting the data by genomic context, we observed that with increasing disease risk methylation increased in CpG dense regions, ‘islands’ and ‘shores’, and decreased at repetitive elements (LINE‐1, LTR, Alu) (Figures 1B and S2).

Next, we sought to identify if there were specific genomic regions of differential methylation that could be used as biomarkers to distinguish between non‐lethal and lethal prostate cancer. Using DMRcate,^17^ we identified 1420 DMRs, with the majority (92%) hypermethylated (increased) in the ‘lethal’ compared to the ‘non‐lethal’ RP samples (Table S2 and Figure 1C). For example, a 1 kb region at the promoter CpG island of the tumour suppressor *DEUP1(CCDC67)* gene^37^ showed significant hypermethylation, with an average 38% methylation increase in the ‘lethal’ compared to ‘non‐lethal’ disease groups (Figure 1D).

To examine the potential functional importance of these DMRs, we determined their location relative to specific genomic features (Figure 1E). Consistent with our initial global methylation analysis of CpG context, we found that most hypermethylated DMRs were located in CpG islands (74%), whereas hypomethylated DMRs were more common in non‐CpG islands regions (75%) (Figure 1E, left panel). Relative to annotated protein‐coding genes, 48% of hypermethylated compared to 14% of hypomethylated DMRs were located at promoter regions, and overall hypermethylated DMRs were located closer to transcription start sites (TSSs) (median distance to TSS hypermethylated DMRs = 745 bp vs. hypomethylated DMRs = 12 799 bp, Mann–Whitney *U*‐test, *p* = 4.6e‐21) (Figure 1E, right panel).

We next examined the overlap between the DMRs and putative regulatory elements, using ChromHMM segmentation data from prostate cell lines^20^: normal PrEC and LNCaP cells. This analysis revealed that the ‘lethal’ hypermethylated DMRs overlapped with more active regulatory regions in PrEC compared to LNCaP (active TSS, flanking active TSS and bivalent enhancer regions) and more silenced regions in the LNCaP compared to PrEC (heterochromatin and quiescent regions) (Figure 1F). This comparison suggests that the hypermethylated DMRs may represent promoters and enhancers that are epigenetically silenced with prostate cancer severity, as is known to occur at tumour suppressor genes during tumourigenesis.^4^ In contrast, the hypomethylated DMRs overlap with more silenced regions in PrEC compared to LNCaP (heterochromatin and quiescent regions) and more active regulatory regions in LNCaP compared to PrEC (active TSS, flanking active TSS and enhancer regions) (Figure S3). The hypomethylated DMRs may therefore represent promoters that are epigenetically activated with prostate cancer severity, as is known to occur at oncogenes during tumourigenesis.^4^ Additionally, WGBS methylation data^7^ from the LNCaP and PrEC cells show that the 1420 DMRs are also highly differentially methylated between these cell lines, with 97% of the hypermethylated DMRs showing an increase in LNCaP compared to PrEC, and 89% of the hypomethylated DMRs showing a decrease in LNCaP compared to PrEC (Figure 1C). Combined with the results from the LNCaP and PrEC chromatin state data, this provides further support for an association between DNA methylation and changes in gene activation with disease progression.

We next examined whether the DMRs were enriched in particular biological pathways through comparison with curated gene sets from the MSigDB. Hypermethylated DMRs were significantly enriched for 298 terms (see Table S3). Notably, within the MSigDB C2 collection, the DMRs demonstrated enrichment for cancer‐associated gene sets, including genes reported as downregulated in prostate cancer and as *de novo* methylated in cancer (Figure 1G). Hypomethylated DMRs were only enriched for one MSigDB term (Table S3), a cancer‐related gene set.

### 3.2 Selection criteria of genomic regions for a prognostic DNA methylation panel

We next sought to test the clinical relevance of the DMRs through the development of a prognostic DNA methylation panel for validation in an independent prostate cancer cohort. We applied stringent selection criteria to identify DMRs showing prognostic potential, resulting in 18 DMRs that we considered suitable for inclusion in the DNA methylation panel (Tables 2 and S4 for full details and methylation values). First, we used the WGBS DMR results to select hypermethylated DMRs with a large mean difference between ‘lethal’ and ‘non‐lethal’ RP samples (≥30%), that also importantly had low methylation levels in the WGBS data from the normal prostate tissue samples (Figure S4A). Next, we used two publicly available DNA methylation microarray (Illumina HM450K) datasets to confirm that, for those DMRs targeted by probes on the microarray, the DMRs were indeed hypermethylated in tumour versus normal tissue (PRAD samples from TCGA Data Portal), and hypomethylated in blood samples (GSE40279 from the NCBI GEO database),^25^ to allow the potential use of the DNA methylation panel in liquid biopsies in the future (Figure S4B). Interestingly, we found highly significant correlations between methylation and expression levels of the nearest‐protein coding gene in the TCGA PRAD samples for the majority of DMRs (Figure S5 and Table S4). We then used our PrEC and LNCaP WGBS^7^ and chromatin state data^20^ to confirm the potential functional relevance of these specific regions in this cellular model of advanced prostate cancer. Here, we required DMRs to also show a large increase in methylation in LNCaP versus PrEC (Figure S4C), and prioritised those DMRs that exhibited a change from an active to a repressed state to indicate the functional importance of the DMR. Full data for DMRs meeting these criteria are shown in Figure S1 and summarised in Table S4. Finally, a review of the literature showed that many of the DMRs were located near to genes with a known role in prostate cancer or other cancer types (Table S4), whereas other DMR genes were novel, thus allowing us to explore both known and novel candidates through our selected 18 regions.

**TABLE 2:** The panel of 18 differentially methylated regions (DMRs) chosen to be validated as prognostic biomarkers

DMR no.	Genomic position (hg19)	Nearest‐protein coding gene	Distance to TSS (bp)	Lethal mean methylation (WGBS) (%)	Non‐lethal mean methylation (WGBS) (%)	Normal tissue mean methylation (WGBS) (%)
1	chr1:208132439‐208132824	*CD34*	47 691	69.23	30.89	4.51
2	chr2:27958210‐27958689	*AC074091.13*	19 610	58.58	16.09	5.32
3	chr3:184243657‐184243936	*EPHB3*	35 635	62.83	23.66	8.94
4	chr4:81118427‐81118588	*PRDM8*	69	70.95	28.67	26.36
5	chr5:10333634‐10334055	*MARCH6*	19 759	59.00	20.00	3.43
6	chr5:115151283‐115152645	*CDO1*	−5	52.55	19.09	3.46
7	chr7:99155673‐99157071	*ZNF655*	0	42.26	14.01	1.66
8	chr8:95246476‐95246871	*CDH17*	16 944	59.10	19.29	11.48
9	chr11:93063135‐93064069	*DEUP1*	0	63.99	25.44	6.97
10	chr12:1906206‐1906676	*CACNA2D4*	−14 209	54.89	12.29	11.75
11	chr12:3862069‐3862497	*CRACR2A*	0	44.67	4.37	4.62
12	chr12:103311054‐103311276	*PAH*	21	64.43	22.12	2.29
13	chr13:53312994‐53313591	*CNMD*	0	69.66	30.95	6.84
14	chr14:90849492‐90850589	*CALM1*	12 256	49.11	3.85	4.56
15	chr15:72564636‐72565252	*PARP6*	0	45.41	9.11	2.02
16	chr17:62773682‐62777796	*LRRC37A3*	−77 948	61.14	28.82	10.69
17	chr22:19742681‐19743728	*TBX1*	497	68.39	29.20	14.29
18	chrX:102000717‐102001518	*BHLHB9*	0	51.15	5.97	1.86

*Note*: For the distance of DMRs to the TSS of the nearest‐protein coding gene, positive values indicate that the DMR lies downstream of the TSS, whilst negative values indicate that the DMR lies upstream of the TSS.

Abbreviations: TSS, transcription start site; WGBS, whole‐genome bisulphite sequencing.

### 3.3 Validation of prognostic DNA methylation panel in an independent RP cohort

We used MBPS^28^ to quantify methylation at the selected 18 regions in FFPE tumour tissue from a well‐characterised, population‐based cohort of 185 patients with localised prostate cancer, with median 15 years follow‐up and ISUP Grade Group ≥2^10^ (see Table 1B for cohort details). Details on the MBPS primer sequences and locations are given in Table S1 and Figure S1. Sequencing data from this panel were processed using *MethPanel*,^31^ which showed that 17 out of 18 regions passed quality control (*CDH17—*DMR #8 had low coverage and so was removed from all downstream analysis). PCR bias was observed to be minimal, with no batch effects between sequencing runs (Figure S6), and extremely high sequencing coverage (averaging ∼100 000 reads) was achieved across all 17 regions (Figure S7 and Table S5). Correlations between DNA methylation at the 17 genomic regions (pairwise *r* ranging from 0.16 to 0.65) were consistently positive but incompletely correlated, indicating that each region may contribute unique information (Figure S8).

### 3.4 Survival analysis

Clinical follow‐up data in the validation cohort, with a median of 15 years (range: 0.8–22 years), showed that 86 patients (46.5%) had a BCR, 25 patients (13.5%) had an MR and 16 patients (8.6%) died of prostate cancer. One patient was missing pre‐operative PSA data, so was excluded from subsequent analysis, leaving *n* = 185 patients. The survival analysis was conducted in three stages, as described below.

#### 3.4.1 Univariable clinicopathological analysis

**FIGURE 2:**
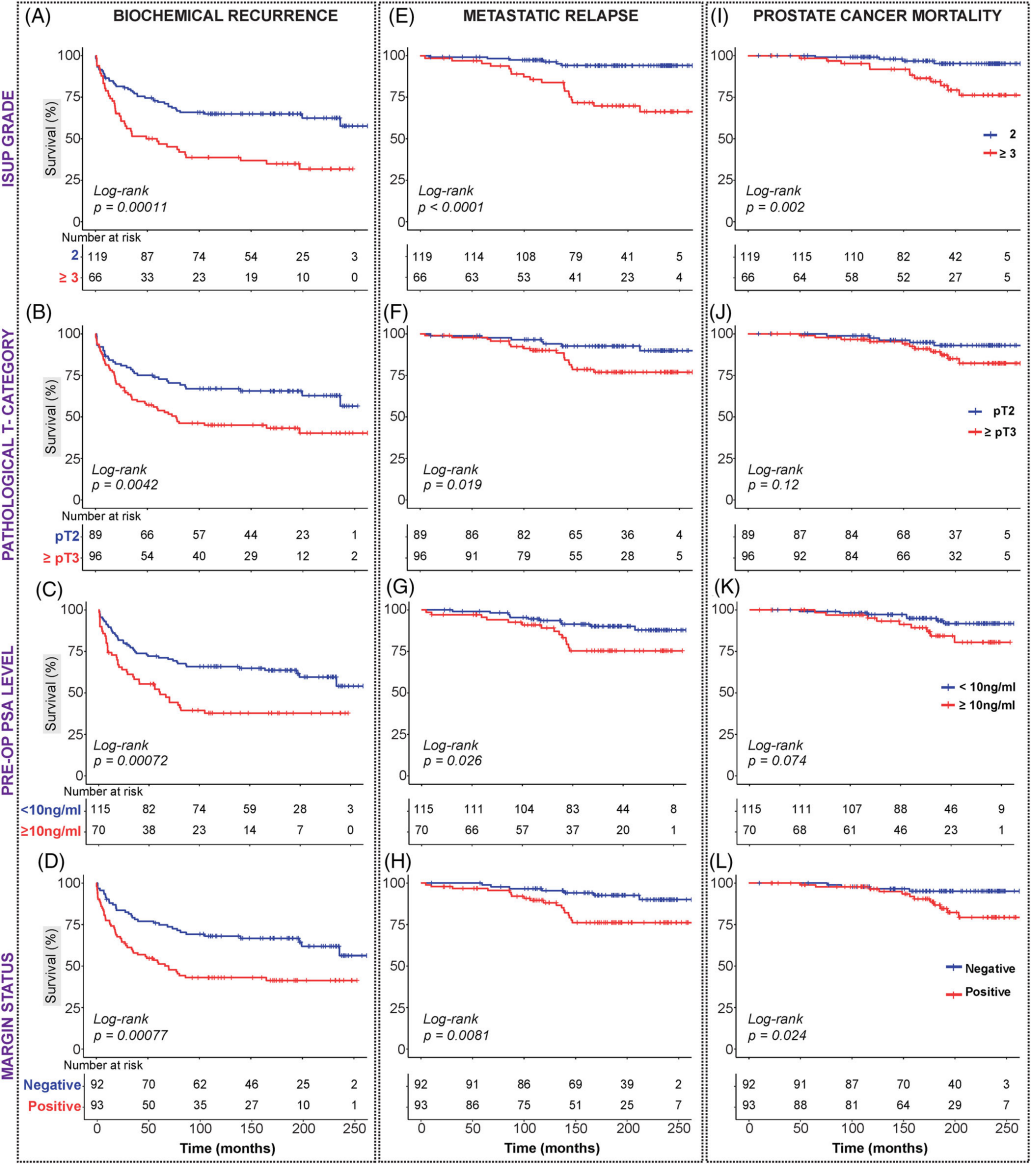
Kaplan–Meier survival curves: clinicopathological factors. Kaplan–Meier survival analysis of four clinicopathological factors: ISUP Grade Group (A, E, I), pathological T‐category (B, F, J), pre‐op prostate‐specific antigen (PSA) level (C, G, K) and margin status (D, H, L) across three endpoints—biochemical recurrence (A–D), metastatic relapse (E–H) and prostate cancer‐specific mortality (I–L).

**FIGURE 3:**
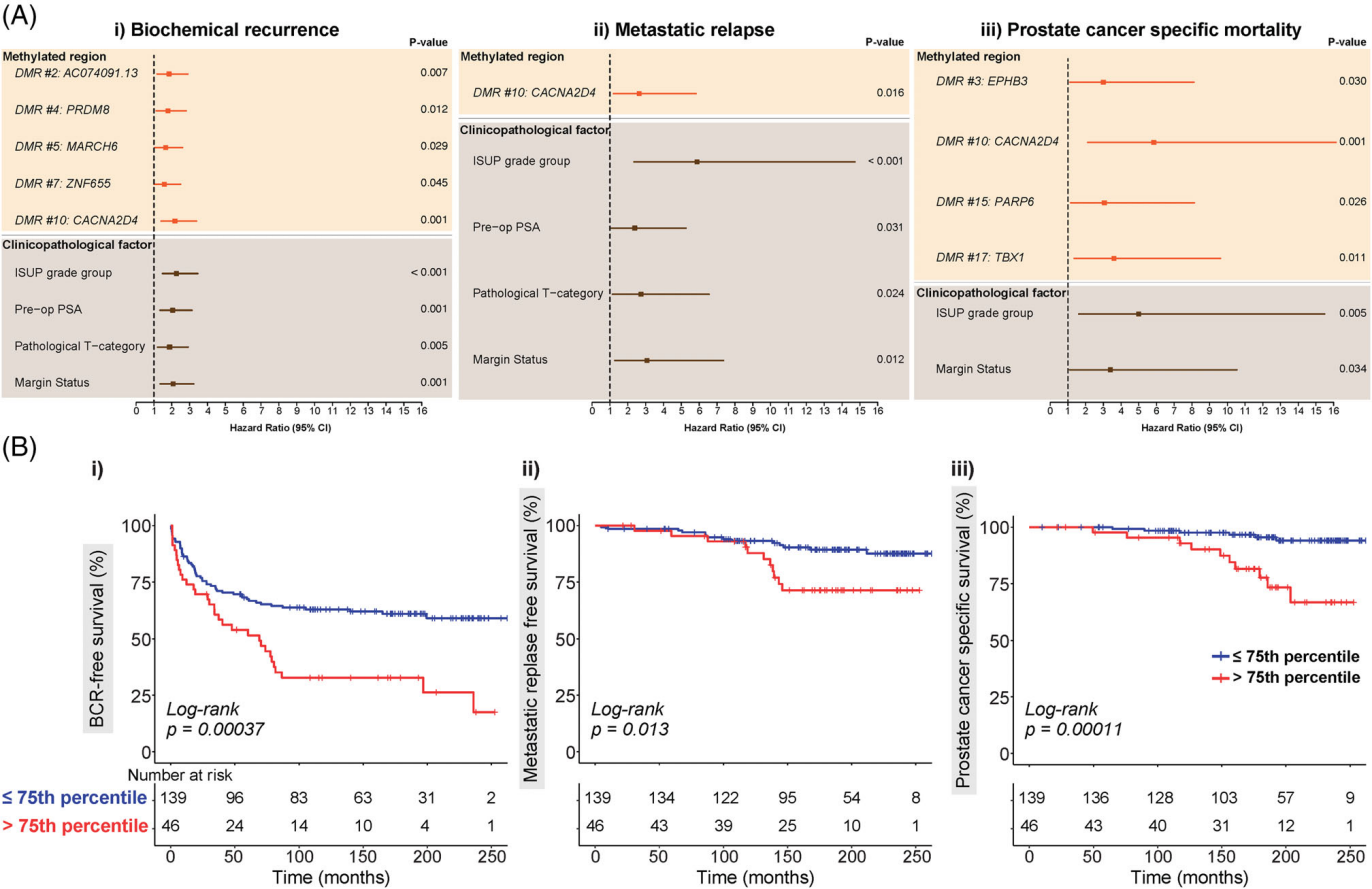
Univariable survival analysis. (A) Univariable Cox regression analysis: Forest plots showing the univariable hazard ratio, 95% confidence interval (CI) and *p*‐value for methylation and clinicopathological variables significant in univariable Cox regression analysis for: (i) biochemical recurrence, (ii) metastatic relapse and (iii) prostate cancer‐specific mortality. Methylated regions are shaded in orange, and clinicopathological factors are shaded in brown. (B) Prognostic potential of *CACNA2D4* (DMR #10): Kaplan–Meier survival curves with endpoints of: (i) biochemical recurrence, (ii) metastatic relapse and (iii) prostate cancer‐specific mortality. Red line indicates higher methylation (>75th percentile) and blue line indicates lower methylation (≤75th percentile).

Log‐rank and Cox regression analyses were performed to evaluate the associations between clinicopathological factors and BCR‐free survival, MR‐free survival and PCSM. Both log‐rank (Figure 2 and Table S6) and univariable Cox regression analyses (Figures 3A and S9 and Table S6) showed that all four routine prognostic clinicopathological factors (ISUP Grade Group, pathological T‐category, pre‐operative PSA level and surgical margin status) were significantly associated with time to BCR (Figures 2A–D and 3Ai), as well as with time to MR (Figures 2E–H and 3Aii). Only ISUP Grade and margin status were identified as significant predictors of prostate cancer death in log‐rank and Cox regression analyses (Figures 2I–L and 3Aiii).

#### 3.4.2 Univariable methylation analysis

To analyse the prognostic capacity of the 17 genomic regions in our methylation panel, the 185 patients in this validation cohort were grouped into low and high methylation groups for each of the regions, based on the 75th percentile value of the MBPS data. Again, log‐rank and univariable Cox regression analyses were performed to examine the relationships between these methylated regions and event‐free survival (Figures 3, S9 and S10 and Table S6). Log‐rank analysis revealed that methylation levels at five genomic regions were associated with BCR‐free survival (*AC074091.13*: *p* = .0066, *CACNA2D4*: *p* = .00037, *PRDM8*: *p* = .011, *MARCH6*: *p* = .027, *ZNF655*: *p* = .043); only *CACNA2D4* was associated with MR‐free survival (*p* = .013) and five regions were associated with PCSM (*CACNA2D4*: *p* = .00011, *EPHB3*: *p* = .023, *PARP6*: *p* = .019, *TBX1*: *p* = .0063, *MARCH6*: *p* = .042) (Figures 3B and S10 and Table S6). As expected (from the analysis of the discovery cohort), higher methylation levels were associated with poorer survival. Similar results were observed in univariable Cox regression analysis (Figures 3A and S9 and Table S6), and notably *CACNA2D4*, which encodes a protein in the voltage‐dependent calcium channel complex,^38^ was a significant predictor of poor outcomes following RP across all three survival endpoints (BCR: *p* = .0005, hazard ratio [HR] = 2.18 [1.4–3.38], Figure 3Ai; MR: *p* = .016, HR = 2.64 [1.2–5.83], Figure 3Aii; PCSM: *p* = .001, HR = 5.84 [2.12–16.13], Figure 3Aiii).

#### 3.4.3 Multivariable analysis

To find the optimal panel of markers with the greatest predictive power, we performed forward selection for multivariable Cox regression, using the four clinicopathological factors and 17 methylated genomic regions. The final multivariable prognostic models for each of the three clinical endpoints (BCR, MR and PCSM) are summarised in Table 3. For BCR, the final model with the most predictive power from SES analysis consisted of *CACNA2D4* (*p* = .003, HR = 1.94 [1.25–3.03]), ISUP Grade Group (*p* = .000, HR = 2.23 [1.45–3.42]) and pre‐operative PSA levels (*p* = .001, HR = 2.08 [1.36–3.2]). For MR, methylation did not add prognostic value, with only ISUP Grade Group (*p* = .000, HR = 5.41 [2.16–13.59]) and margin status (*p* = .028, HR = 2.67 [1.11–6.41]) in the final model. For PCSM, the final multivariable prognostic model consisted of *CACNA2D4* (*p* = .001, HR = 5.33 [1.93–14.73]) and ISUP Grade Group (*p* = .009, HR = 4.53 [1.46–14.07]). Harrell's *C*‐indices show that the addition of *CACNA2D4* methylation improved the predictive accuracy compared to the model containing only the SES‐derived clinicopathological markers (ISUP Grade and pre‐operative PSA levels) in predicting BCR (*C*‐index: 0.680 vs. 0.649) (Table 3A). Notably, the SES‐derived model of *CACNA2D4* and ISUP Grade was a better predictor of PCSM as compared to ISUP Grade alone (*C*‐index: 0.779 vs. 0.684) (Table 3C). In a complementary approach, we applied a time‐dependent ROC analysis to calculate AUC for survival at 1, 5, 10 and 15 years post‐RP. Again at all time points assessed, the models had a higher accuracy with *CACNA2D4* methylation included in the model (Table S7). For example, for PCSM survival at 5 years post‐RP, we observed a clinically relevant increase in AUC from 0.69 with ISUP Grade Group alone to 0.78 after including *CACNA2D4* methylation. Finally, to ensure *CACNA2D4* results were not biased by between‐patient variability in PCR sequencing read depth, we performed bioinformatic downsampling of the *CACNA2D4* sequencing data and repeated the survival analyses. Results remained similar across all sequencing coverage levels (Tables S8 and S9).

**TABLE 3:** Results of multivariable Cox regression analyses in validation cohort (*n* = 185), showing models with the greatest predictive power selected using the Statistically Equivalent Signatures (SES) feature selection algorithm (i) with and (ii) without methylation measurements included as input variables

		(i) Clinicopathological and methylation variables			(ii) Clinicopathological variables only		
Variable	Thresholds	HR (95% CI)	*p*‐Value	*C*‐index	HR (95% CI)	*p*‐Value	*C*‐index
(A) Biochemical recurrence							
*CACNA2D4* (DMR #10)	≤75th percentile versus >75th percentile	1.94 (1.25–3.03)	.003	0.681			
Pathological ISUP Grade Groups	2 versus 3–5	2.23 (1.45–3.42)	.000		2.40 (1.57–3.68)	.000	0.649
Pre‐operative PSA	<10 ng/ml versus ≥10 ng/ml	2.08 (1.36–3.2)	.001		2.19 (1.43–3.37)	.000	
(B) Metastatic relapse							
Pathological ISUP Grade Groups	2 versus 3–5	5.41 (2.16–13.59)	.000	0.760			
Margin status	Negative versus positive	2.67 (1.11–6.41)	.028				
(C) Prostate cancer‐specific mortality							
*CACNA2D4* (DMR #10)	≤75th percentile versus >75th percentile	5.33 (1.93–14.73)	.001	0.779			
Pathological ISUP Grade Groups	2 versus 3–5	4.53 (1.46–14.07)	.009		4.99 (1.61–15.49)	.005	0.684

Abbreviations: CI, confidence interval; DMR, differentially methylated region; HR, hazard ratio; PSA, prostate‐specific antigen.

### 3.5 Characterisation of the *CACNA2D4* DMR

The most promising methylation biomarker identified in our study is a region within the *CACNA2D4* gene, which overlaps exon 35 of the main protein‐coding isoform of the gene, 14 kb downstream of the TSS (Figure S11A). Our original comparison with PrEC and LNCaP data indicated that, although distant from the gene promoter, the DMR overlaps a regulatory DNA element: defined as a hypomethylated, active regulatory region in normal prostate cancer cells that becomes hypermethylated and assumes a weaker active chromatin state in the metastatic cancer cell line (Table S4 and Figure S1J). In support of the region's regulatory importance, we used data from the GeneHancer ‘Double Elite’ list^21^ to confirm that the DMR overlaps a validated promoter/enhancer region (GeneHancer ID: GH12J001795). Next, we used the Double Elite list to identify the regulatory element's likely target gene. Two or more sources of evidence showed that there was a high likelihood of interaction between this region and the *CACNA2D4* gene promoter (Figure S11A). The methylation status of a regulatory region can impact long‐range interactions,^39^ leading us to hypothesise that the differential methylation we observed at this region may affect its interaction with the *CACNA2D4* promoter, and thus its expression. Consistent with this, data from TCGA show that *CACNA2D4* expression is significantly decreased in tumour versus normal tissue (Figure S11B); however, we were unable to assess the corresponding methylation difference in the same cohort as the HM450K platform used by TCGA does not have probes targeting the *CACNA2D4* DMR. Taken together, these data suggest that DNA methylation at the *CACNA2D4* DMR may play a role in long‐range transcriptional regulation of the *CACNA2D4* gene.

## 4 DISCUSSION

Prostate cancer is a highly heterogeneous disease. Current risk stratification tools based on standard clinicopathological variables provide some degree of predictive ability. Advances in high‐throughput genomic and RNA sequencing has led to the development of several novel tissue‐based biomarkers that can improve prostate cancer prognosis to aid disease management, including commercialised gene expression prognostic biomarker tests, such as Decipher (GenomeDx Biosciences, Vancouver, British Columbia, Canada) and Prolaris (Myriad Genetics, Salt Lake City, UT, USA), which both require RNA from prostate tissue.^40^ However, DNA methylation biomarkers could further improve disease prognosis, especially given their potential to be developed for liquid biopsy, such as the FDA approved Epi proColon test for colorectal cancer; based on PCR detection of methylated *SEPT9* (septin 9) in cell‐free circulating DNA shed from tumours into the bloodstream.^41^

DNA methylation is a promising prostate cancer DNA biomarker because it is one of the earliest molecular changes to occur in tumourigenesis and is a more stable molecule than RNA,^42^ allowing it to be measured even in low input, degraded clinical tissue, including FFPE tissue.^28^ However, to date the discovery of prognostic DNA methylation markers has been limited by the technologies and the type of cohorts used.^6^ Even the landmark TCGA prostate cancer study had an average follow‐up time of just 2 years after RP.^43^ Short follow‐up time means that many studies of prostate cancer prognosis use BCR as an indicator of aggressive disease.^6^ However, the ICECaP consortium has identified that MR, not BCR, is the best surrogate for prostate cancer‐specific death.^44^ In this study, we applied single‐base resolution methylation sequencing technologies to two independent prostate cancer cohorts with long‐term follow‐up clinical data (median: >15 years), allowing analysis of the association between DNA methylation alterations throughout the prostate cancer genome and PCSM.

WGBS profiling of the discovery cohort provided new insights into the associations between methylation and prostate cancer risk, and identified a suite of candidate DNA methylation prognostic biomarkers. Initial analysis with PCA showed that the first principal component (which explains the largest proportion of variance in the methylation data) showed separation not only between normal and tumour samples, but also between tumours from patients with lethal and non‐lethal disease. Sub‐setting the data according to genomic context revealed locus‐specific dynamics, with methylation at CpG‐rich islands and shores increasing with the aggressiveness of disease, whereas methylation at repetitive elements showed the opposite association. These results are consistent with the known regional DNA hypermethylation that occurs at promoter CpG islands in cancer, and with the studies of promoter CpG islands of candidate genes that have reported increasing methylation levels with disease progression.^4^ Conversely, repetitive elements, making up ∼45% of the genome, are known to be highly methylated in normal tissue, but lose methylation during tumourigenesis,^45^ which can result in the activation of transposable elements leading to potential mutagenesis.^46^ Furthermore, a recent study of the repetitive element LINE‐1 in prostate cancer showed evidence of increasing loss of methylation with disease progression, that was associated with patient survival.^47^

In the discovery cohort, our analysis of WGBS data identified more than 1000 regions showing methylation changes between the lethal and non‐lethal patient groups, of which the majority were in promoter and CpG island regions. Promoter CpG island methylation is typically inversely correlated with expression of the same gene. As the majority of our DMRs were hypermethylated, this suggests that these methylation changes may play a role in gene silencing. Consistent with this, gene ontology analysis of the genes associated with our DMRs identified enrichment for relevant gene sets including the MSigDB ‘LIU_PROSTATE_CANCER_DN’ set; a list of genes identified as downregulated in human prostate cancer compared to benign tissue.^48^

We selected top‐ranked lethal DMRs for validation in a large, independent clinical cohort. Interestingly, a number of these top‐ranked DMRs occur at the CpG island promoter of genes previously associated with prostate cancer progression (see Table S4 for full details). Amongst these is *Calcium Release Activated Channel Regulator 2A* (*CRACR2A*), which encodes a calcium‐binding protein that is implicated in innate immune response.^49^ A recent study also reported hypermethylation of *CRACR2A* in prostate cancer tissue from men with metastatic‐lethal disease, at a region overlapping the promoter DMR identified in our study. Hypomethylation of this region was also associated with vigorous physical activity in the year before RP, leading the authors to conclude that *CRACR2A* methylation could mediate the link between physical activity and metastatic‐lethal progression.^50^ Another study identified the exact same region of *CRACR2A* (listed as *EFCAB4B*) as the top‐ranking DMR hypermethylated in breast cancer patients resistant to endocrine therapy, with a strong negative correlation with gene expression, and hypothesise that the observed methylation change may be regulating immune/inflammatory alterations in the tumour microenvironment.^49^ Another top‐ranked lethal DMR, *Cysteine Dioxygenase Type 1* (*CDO1*), is a potential tumour suppressor gene, which has shown promoter hypermethylation and gene silencing in a range of different cancers^51^ and notably showed that increased promoter methylation with BCR‐free survival in prostate cancer patients following RP.^52^ *T‐Box Transcription Factor 1* (*TBX1*), a gene encoding a developmental transcription factor and implicated in retinoic acid signalling,^53^ has also been associated with prostate cancer risk. A recent meta‐analysis of 87 040 individuals (43 303 prostate cancer cases and 43 737 controls) identified an intronic single‐nucleotide polymorphism in the *TBX1* gene that was significantly associated with prostate cancer in both European and Japanese populations.^54^ A study using a comprehensive, single‐base resolution technique, Enhanced Reduced Representation Bisulfite Sequencing, to profile DNA methylation in benign prostate, prostate cancer and castrate resistant prostate cancer tissue identified a region in the first intron of the *TBX1* gene that showed increasing levels of methylation with disease severity, together with increased gene expression.^53^

To validate the prognostic utility of the top‐ranked 18 lethal DMRs, we used targeted MBPS^28^ in an independent cohort to test the association between the lethal DMRs and each of the survival endpoints: BCR, MR and PCSM. Log‐rank analysis revealed five DMRs associated with BCR, one DMR with MR and five with PCSM. Given the growing recognition that BCR may not be a good predictor of PCSM, it was interesting to note that a different set of DMRs was associated with these two survival endpoints. *CACNA2D4*, however, was significantly associated with all three endpoints. It was also the only DMR to be selected in the final multivariable models for BCR and PCSM, as a significant independent prognostic variable, as measured by Harrell's *C*‐index. Inclusion of *CACNA2D4* methylation in multivariable models substantially improved the *C*‐index for predicting PCSM from 0.684 with clinicopathological variables alone to 0.779. This *C*‐index increase is comparable to the improvements afforded to survival models (using standard clinicopathological variables) by the inclusion of data from commercialised gene expression prognostic biomarker tests, such as Decipher and Prolaris. For example, a 2015 study reported that the cell cycle progression score used by Prolaris improved the survival model prediction of 10 year PCSM from a *C*‐index of 0.74–0.78.^55^ For comparison, the inclusion of the Decipher Genomic Classifier signature, measured in RP tissue, increased the survival model *C*‐index for predicting MR at 10 years post‐RP from 0.77 to 0.87.^56^ Whilst another study using biopsy tissue reported that Decipher improved the *C*‐index from 0.60 to 0.71.^57^ However, it should be noted that these Decipher studies were focused on prediction of MR, and did not calculate a *C*‐index for multivariable models of PCSM prediction; therefore, we cannot directly compare these studies with our *CACNA2D4* multivariable model of PCSM.

*CACNA2D4* encodes a protein in the voltage‐dependent calcium channel complex, which mediates the influx of calcium ions into the cell.^38^ Evidence of a role for *CACNA2D4* in cancer is limited (see Table S4).^58^, ^59^, ^60^ We found that the *CACNA2D4* DMR overlaps a regulatory element that is known to target the *CACNA2D4* promoter, suggesting that methylation at this DMR may play a role in long‐range transcriptional regulation of the *CACNA2D4* gene; however, further work, using techniques such as Hi‐C and CRISPR interference,^21^, ^61^ is necessary to fully assess the regulatory mechanisms of this region. Of note, the *CACNA2D4* DMR is not covered by the commonly used methylation microarrays, which may also explain why *CACNA2D4* methylation has not been identified as a prognostic biomarker before.

One of the main strengths of the current study is the long‐term follow up (>15 years), which allowed for the clinically important endpoints of MR and PCSM to manifest. However, the longevity of the study means that it ran over a period in which clinical practice changed: widespread PSA screening was introduced in Australia in 1995, which led to downward stage migration at diagnosis.^62^ The majority of patients in the discovery cohort were diagnosed in the early 1990s, in the pre‐PSA screening era when more men were diagnosed with advanced cancer, whilst the validation cohort patients were diagnosed from 1997 onwards, in the post‐PSA screening era. The change in clinical diagnostics between the cohorts is a possible drawback in the study design, although we are encouraged that many of the DMRs identified in the discovery cohort did provide prognostic value in the validation cohort, as a cohort which reflects contemporary diagnosis and treatment algorithms.

## 5 CONCLUSIONS

Our findings provide a promising foundation for larger prospective randomised studies to validate our novel panel of epigenetic biomarkers, including the potential predictive value of the *CACNA2D4* locus. Future mechanistic studies will determine if any of these epigenetic biomarkers identify early disruption of key regulatory pathways that lead to prostate cancer metastases and death.

## CONFLICT OF INTEREST

The authors declare they have no conflicts of interest.

## Supporting information

Supporting Information

## Data Availability

The data generated as part of this study are available from NCBI Gene Expression Omnibus (GEO) (www.ncbi.nlm.nih.gov/geo) under accession number GSE158927. Reviewers can access using the following link and password (‘token’): https://www.ncbi.nlm.nih.gov/geo/query/acc.cgi?acc=GSE158927 Token: knihouiytzevxgz
