# Supplementary material for: Comprehensive methylome sequencing reveals prognostic epigenetic biomarkers for prostate cancer mortality
Source: Clin Transl Med. 2022 Sep 30;12(10):e1030. doi: 10.1002/ctm2.1030 (PMC9523674; doi:10.1002/ctm2.1030)
Supplement: Supplementary file 1 — Supporting Information [file CTM2-12-e1030-s001.zip › Supplementary_Figures.pdf]

A) *CD34* (DMR #1)

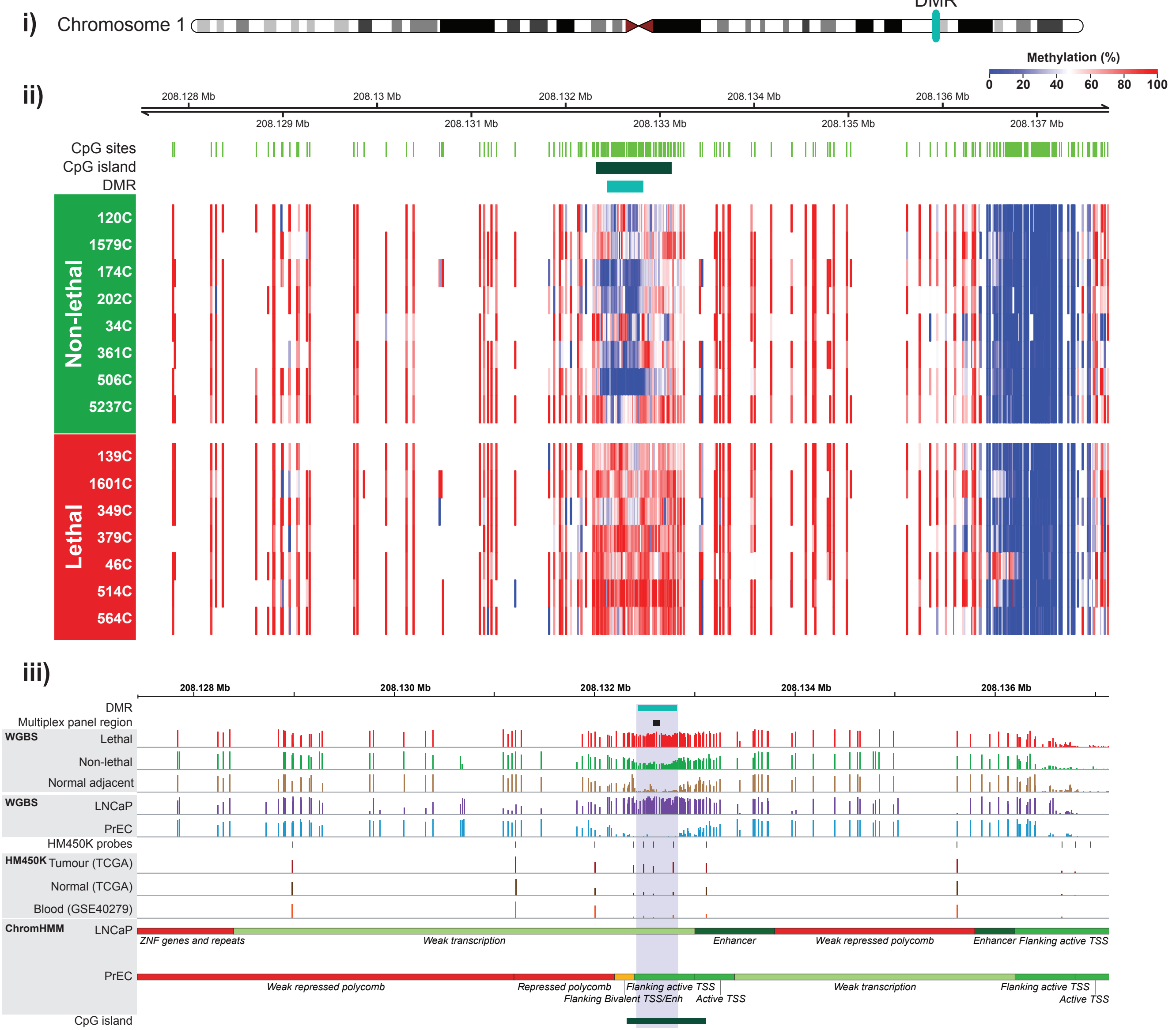
i)
Chromosome 1
DMR
Methylation (%)
0 20 40 60 80 100
ii)
208.128 Mb
208.13 Mb
208.132 Mb
208.134 Mb
208.136 Mb
208.129 Mb
208.131 Mb
208.133 Mb
208.135 Mb
208.137 Mb
CpG sites
CpG island
DMR
Non-lethal
120C
1579C
174C
202C
34C
361C
506C
5237C
Lethal
139C
1601C
349C
379C
46C
514C
564C
iii)
208.128 Mb
208.130 Mb
208.132 Mb
208.134 Mb
208.136 Mb
DMR
Multiplex panel region
WGBS
Lethal
Non-lethal
Normal adjacent
WGBS
LNCaP
PrEC
HM450K probes
HM450K
Tumour (TCGA)
Normal (TCGA)
Blood (GSE40279)
ChromHMM
LNCaP
ZNF genes and repeats
Weak transcription
Enhancer
Weak repressed polycomb
Enhancer Flanking active TSS
PrEC
Weak repressed polycomb
Repressed polycomb
Flanking active TSS
Flanking Bivalent TSS/Enh
Active TSS
Weak transcription
Flanking active TSS
Active TSS
CpG island

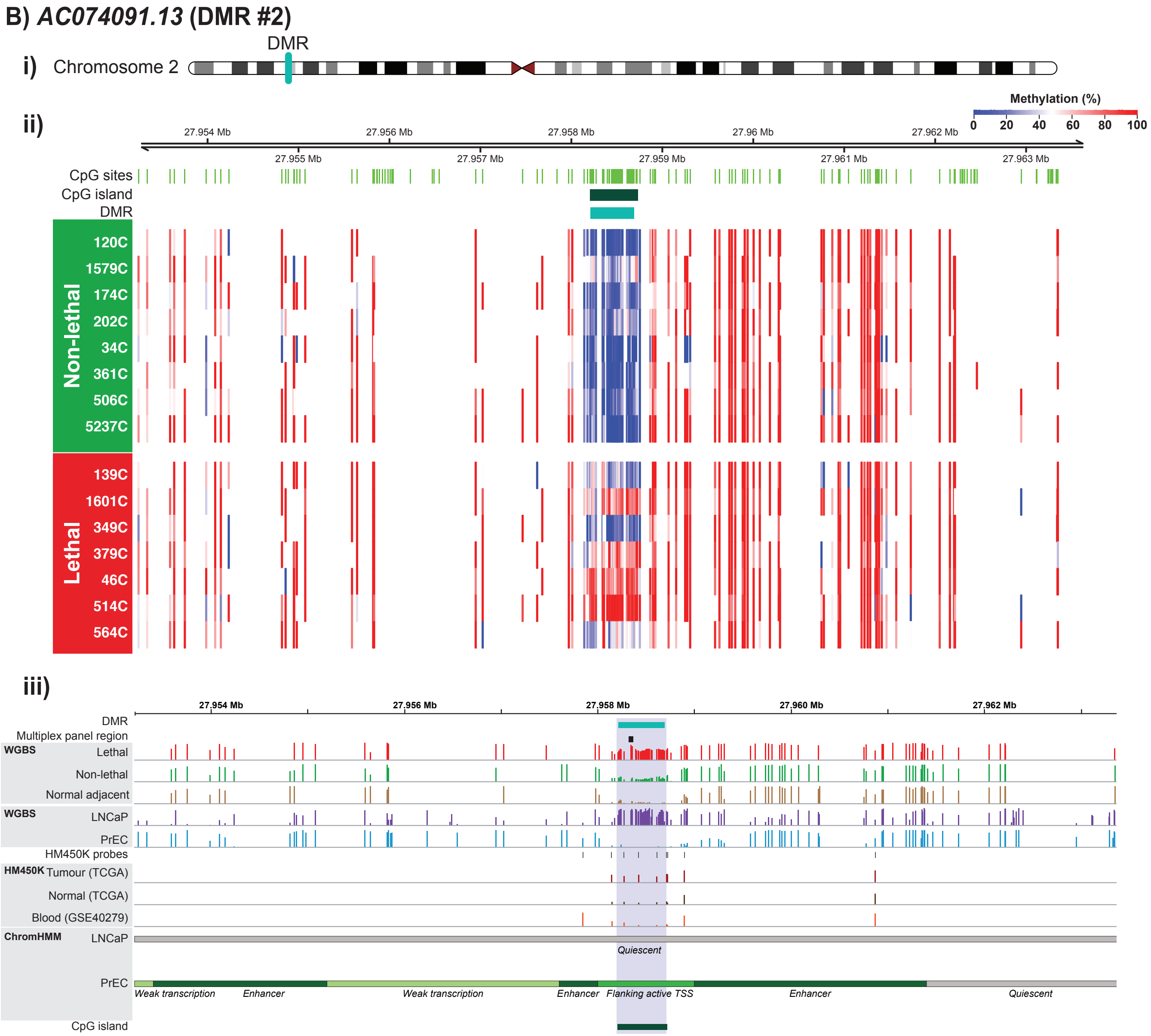

B) AC074091.13 (DMR #2)
i) Chromosome 2
DMR
ii)
Methylation (%)
0 20 40 60 80 100
27.954 Mb
27.955 Mb
27.956 Mb
27.957 Mb
27.958 Mb
27.959 Mb
27.96 Mb
27.961 Mb
27.962 Mb
27.963 Mb
CpG sites
CpG island
DMR
Non-lethal
120C
1579C
174C
202C
34C
361C
506C
5237C
Lethal
139C
1601C
349C
379C
46C
514C
564C
iii)
27.954 Mb
27.956 Mb
27.958 Mb
27.960 Mb
27.962 Mb
DMR
Multiplex panel region
WGBS
Lethal
Non-lethal
Normal adjacent
WGBS
LNCaP
PrEC
HM450K probes
HM450K
Tumour (TCGA)
Normal (TCGA)
Blood (GSE40279)
ChromHMM
LNCaP
Quiescent
PrEC
Weak transcription
Enhancer
Weak transcription
Enhancer
Flanking active TSS
Enhancer
Quiescent
CpG island

C) *EPHB3* (DMR #3)

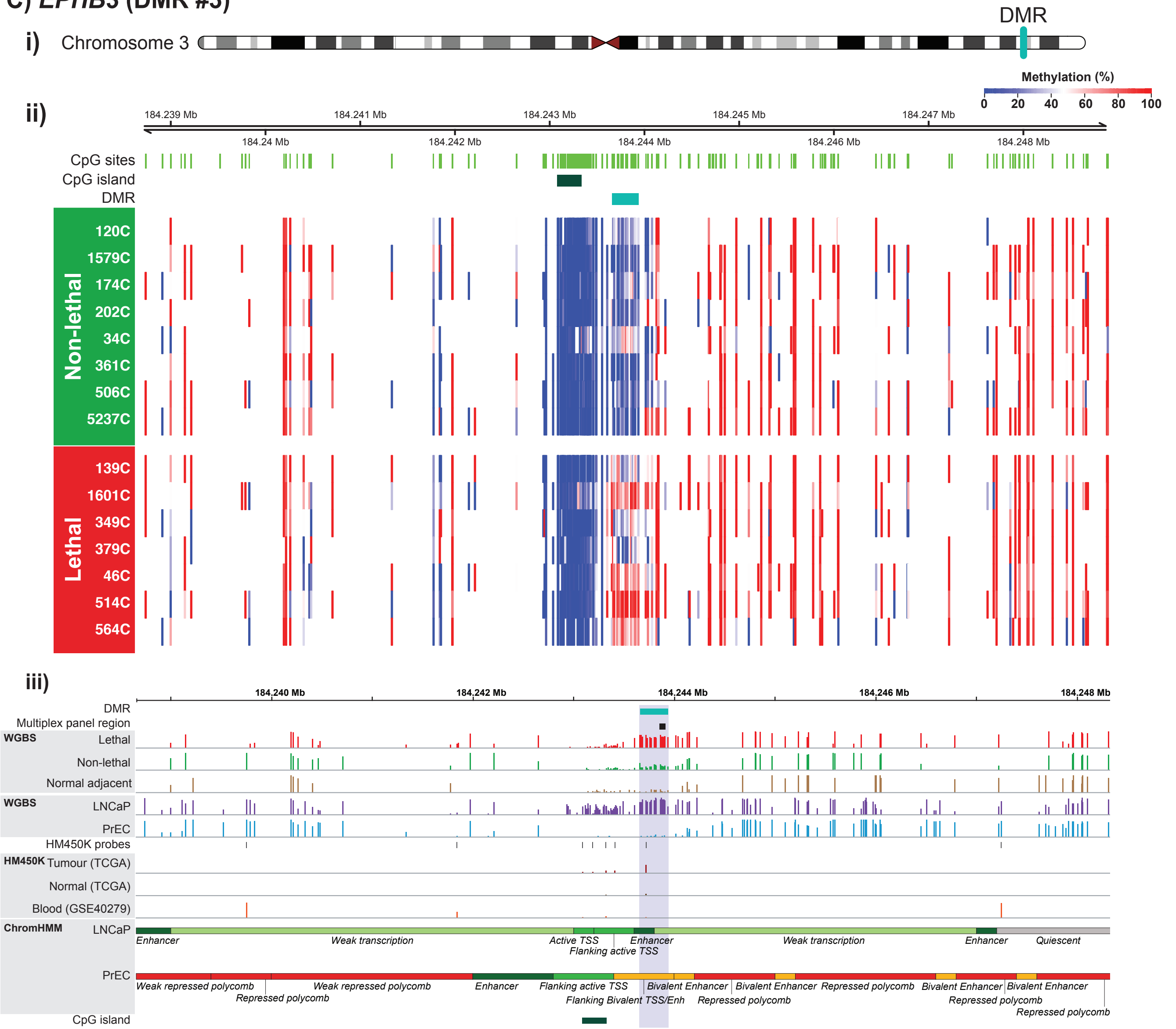

i)
Chromosome 3
DMR
Methylation (%)
0 20 40 60 80 100
ii)
184.239 Mb
184.241 Mb
184.243 Mb
184.245 Mb
184.247 Mb
184.24 Mb
184.242 Mb
184.244 Mb
184.246 Mb
184.248 Mb
CpG sites
CpG island
DMR
Non-lethal
120C
1579C
174C
202C
34C
361C
506C
5237C
Lethal
139C
1601C
349C
379C
46C
514C
564C
iii)
184.240 Mb
184.242 Mb
184.244 Mb
184.246 Mb
184.248 Mb
DMR
Multiplex panel region
WGBS
Lethal
Non-lethal
Normal adjacent
WGBS
LNCaP
PrEC
HM450K probes
HM450K
Tumour (TCGA)
Normal (TCGA)
Blood (GSE40279)
ChromHMM
LNCaP
Enhancer
Weak transcription
Active TSS
Flanking active TSS
Enhancer
Weak transcription
Enhancer
Quiescent
PrEC
Weak repressed polycomb
Repressed polycomb
Weak repressed polycomb
Enhancer
Flanking active TSS
Flanking Bivalent TSS/Enh
Bivalent Enhancer
Repressed polycomb
Bivalent Enhancer
Repressed polycomb
Bivalent Enhancer
Repressed polycomb
Bivalent Enhancer
Repressed polycomb
CpG island

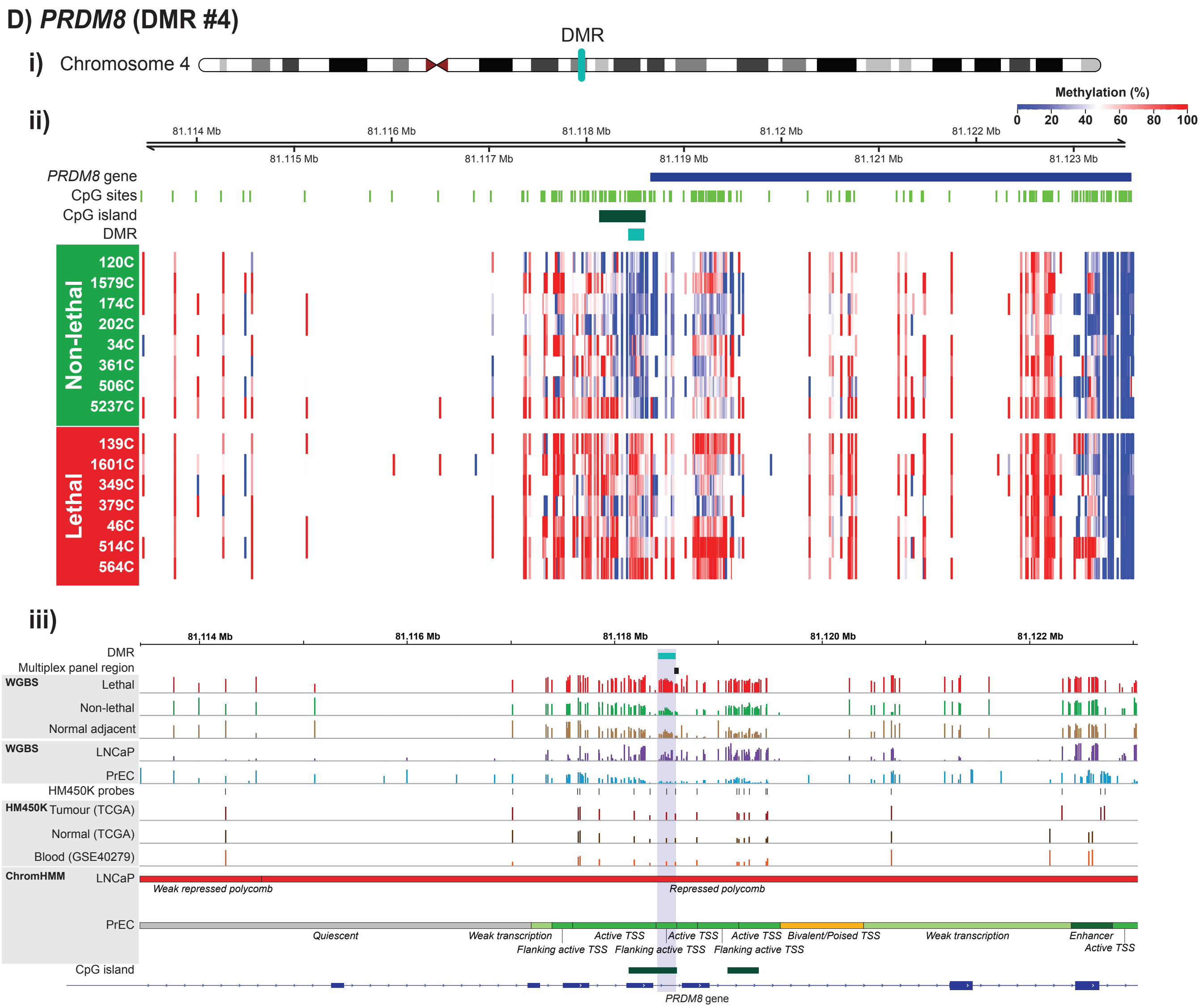

D) PRDM8 (DMR #4)
i) Chromosome 4
DMR
ii)
Methylation (%)
0 20 40 60 80 100
81.114 Mb 81.115 Mb 81.116 Mb 81.117 Mb 81.118 Mb 81.119 Mb 81.12 Mb 81.121 Mb 81.122 Mb 81.123 Mb
PRDM8 gene
CpG sites
CpG island
DMR
Non-lethal
120C 1579C 174C 202C 34C 361C 506C 5237C
Lethal
139C 1601C 349C 379C 46C 514C 564C
iii)
81.114 Mb 81.116 Mb 81.118 Mb 81.120 Mb 81.122 Mb
DMR
Multiplex panel region
WGBS
Lethal
Non-lethal
Normal adjacent
WGBS
LNCaP
PrEC
HM450K probes
HM450K
Tumour (TCGA)
Normal (TCGA)
Blood (GSE40279)
ChromHMM
LNCaP
Weak repressed polycomb
Repressed polycomb
PrEC
Quiescent
Weak transcription
Active TSS
Active TSS
Active TSS
Bivalent/Poised TSS
Weak transcription
Enhancer
Flanking active TSS
Flanking active TSS
Flanking active TSS
Active TSS
CpG island
PRDM8 gene

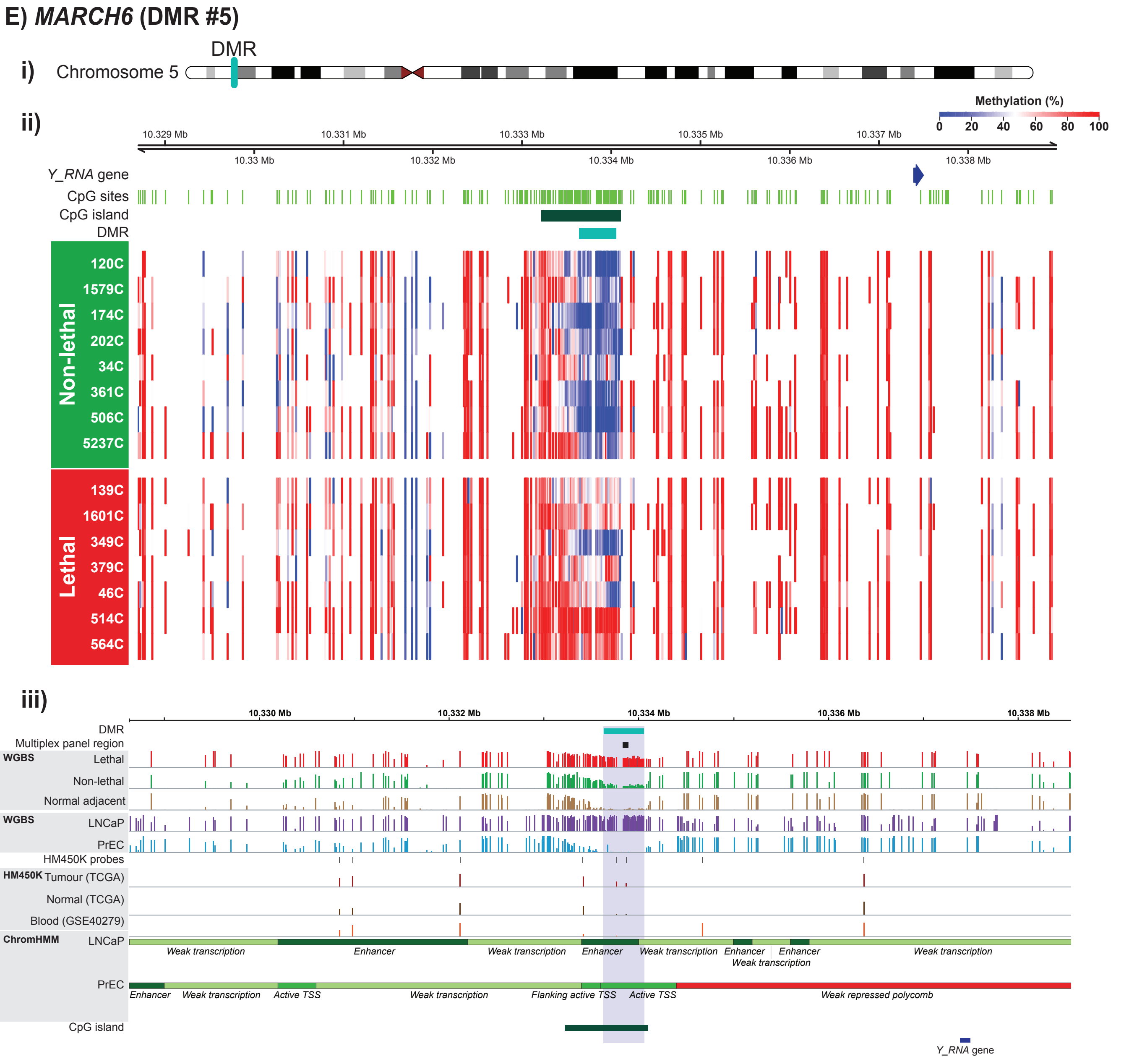

E) MARCH6 (DMR #5)
i)
Chromosome 5
DMR
ii)
Methylation (%)
0 20 40 60 80 100
10.329 Mb
10.331 Mb
10.333 Mb
10.335 Mb
10.337 Mb
10.33 Mb
10.332 Mb
10.334 Mb
10.336 Mb
10.338 Mb
Y_RNA gene
CpG sites
CpG island
DMR
Non-lethal
120C
1579C
174C
202C
34C
361C
506C
5237C
Lethal
139C
1601C
349C
379C
46C
514C
564C
iii)
10,330 Mb
10,332 Mb
10,334 Mb
10,336 Mb
10,338 Mb
DMR
Multiplex panel region
WGBS
Lethal
Non-lethal
Normal adjacent
WGBS
LNCaP
PrEC
HM450K probes
HM450K
Tumour (TCGA)
Normal (TCGA)
Blood (GSE40279)
ChromHMM
LNCaP
Weak transcription
Enhancer
Weak transcription
Enhancer
Weak transcription
Enhancer
Enhancer
Weak transcription
Weak transcription
PrEC
Enhancer
Weak transcription
Active TSS
Weak transcription
Flanking active TSS
Active TSS
Weak repressed polycomb
CpG island
Y_RNA gene

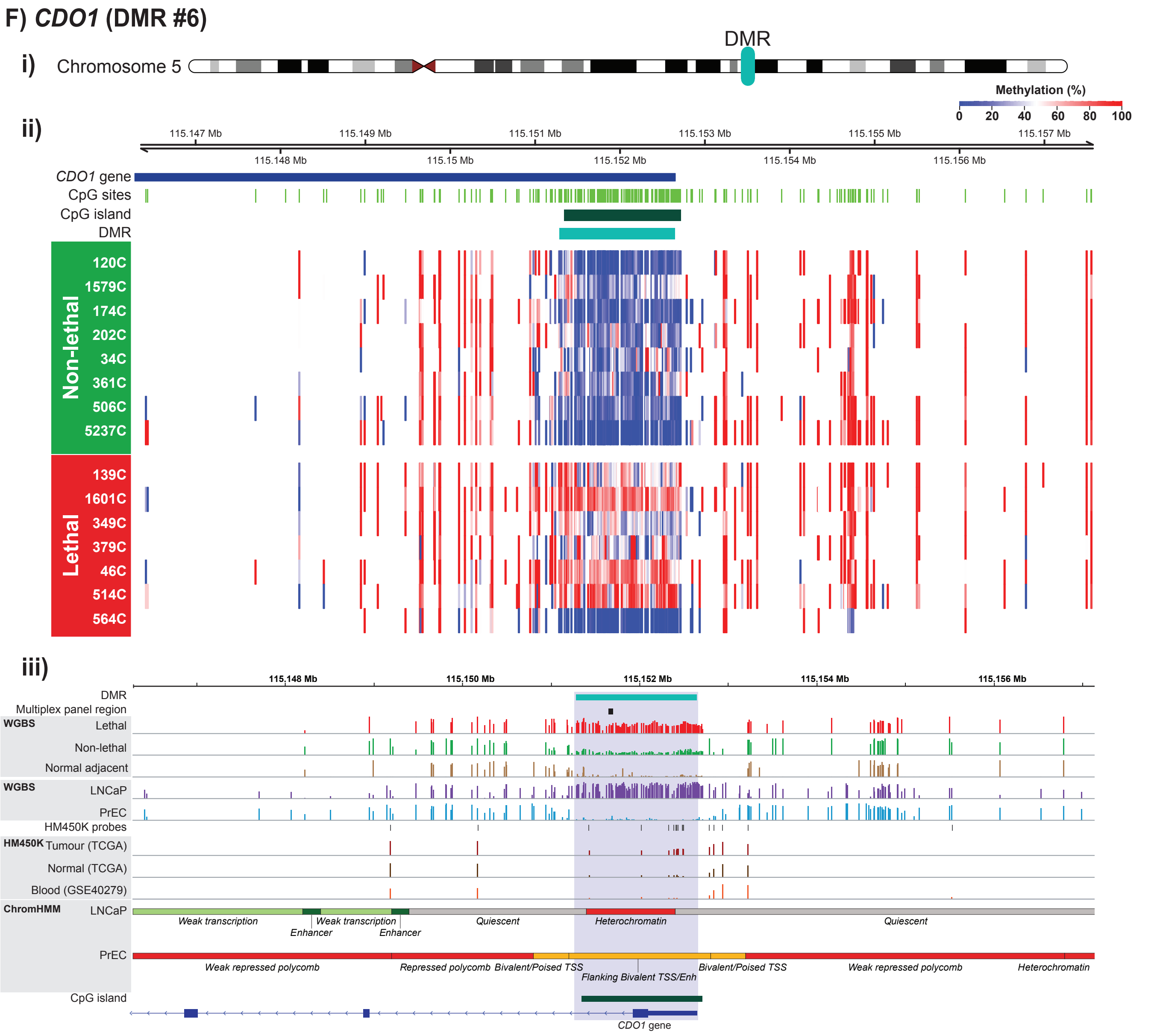
F) CDO1 (DMR #6)
i) Chromosome 5
DMR
Methylation (%)
0 20 40 60 80 100
ii)
115.147 Mb 115.149 Mb 115.151 Mb 115.153 Mb 115.155 Mb 115.157 Mb
115.148 Mb 115.15 Mb 115.152 Mb 115.154 Mb 115.156 Mb
CDO1 gene
CpG sites
CpG island
DMR
Non-lethal
120C
1579C
174C
202C
34C
361C
506C
5237C
Lethal
139C
1601C
349C
379C
46C
514C
564C
iii)
115.148 Mb 115.150 Mb 115.152 Mb 115.154 Mb 115.156 Mb
DMR
Multiplex panel region
WGBS
Lethal
Non-lethal
Normal adjacent
WGBS
LNCaP
PrEC
HM450K probes
HM450K
Tumour (TCGA)
Normal (TCGA)
Blood (GSE40279)
ChromHMM
LNCaP
Weak transcription
Enhancer
Weak transcription
Enhancer
Quiescent
Heterochromatin
Quiescent
PrEC
Weak repressed polycomb
Repressed polycomb
Bivalent/Poised TSS
Flanking Bivalent TSS/Enh
Bivalent/Poised TSS
Weak repressed polycomb
Heterochromatin
CpG island
CDO1 gene

G) *ZNF655* (DMR #7)

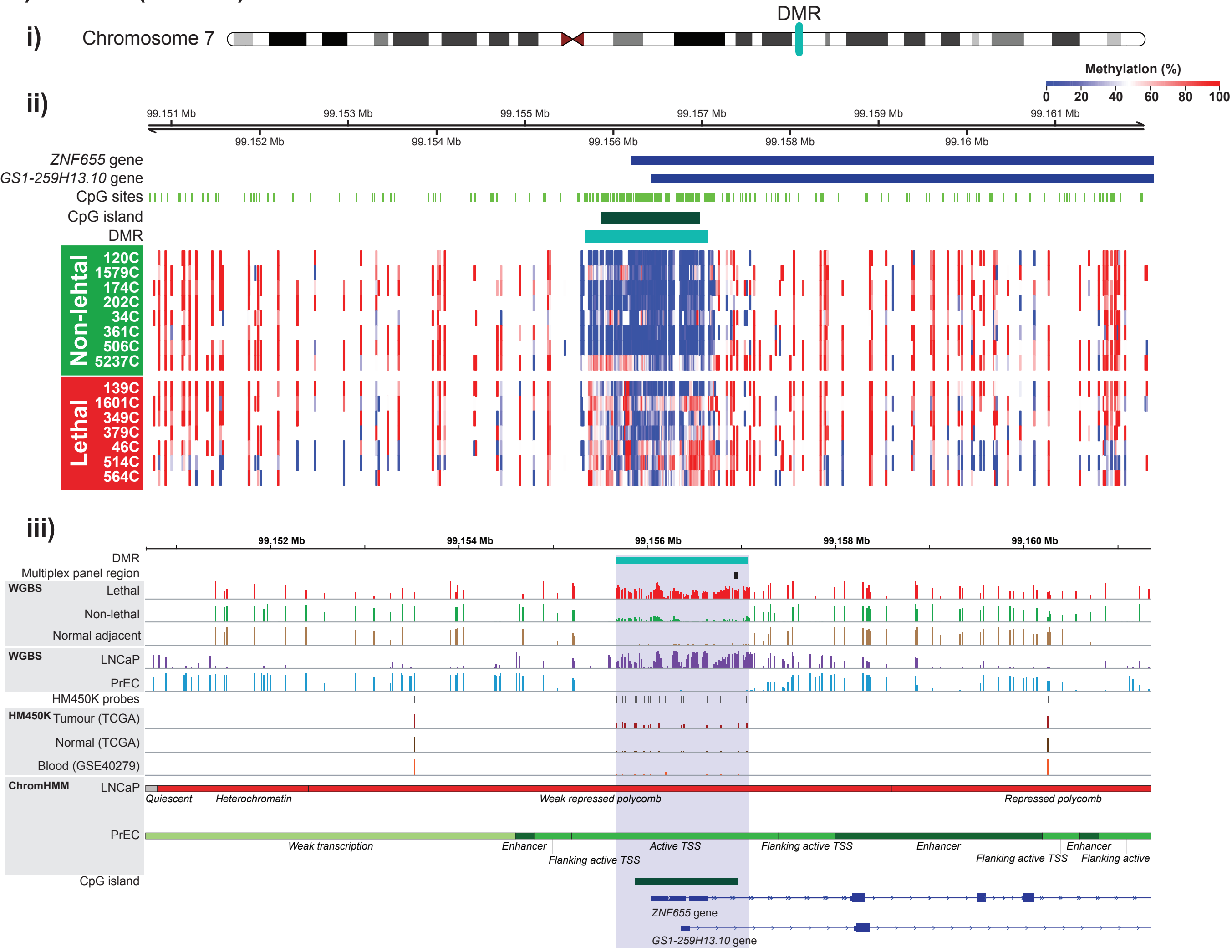

H) *CDH17* (DMR #8)

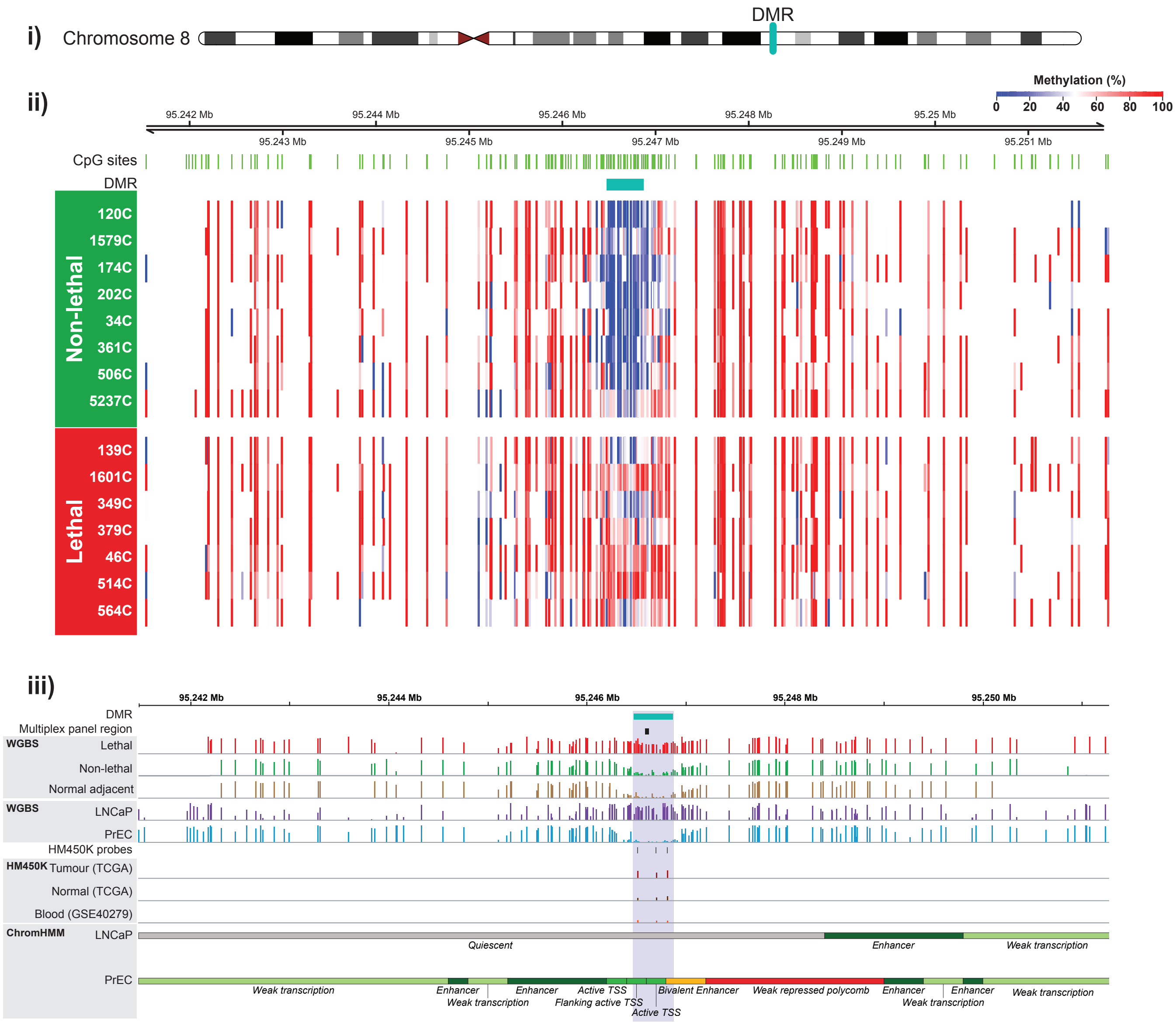
i) Chromosome 8
DMR
ii)
Methylation (%)
0 20 40 60 80 100
95.242 Mb
95.244 Mb
95.246 Mb
95.248 Mb
95.25 Mb
95.243 Mb
95.245 Mb
95.247 Mb
95.249 Mb
95.251 Mb
CpG sites
DMR
Non-lethal
120C
1579C
174C
202C
34C
361C
506C
5237C
Lethal
139C
1601C
349C
379C
46C
514C
564C
iii)
95.242 Mb
95.244 Mb
95.246 Mb
95.248 Mb
95.250 Mb
DMR
Multiplex panel region
WGBS
Lethal
Non-lethal
Normal adjacent
WGBS
LNCaP
PrEC
HM450K probes
HM450K
Tumour (TCGA)
Normal (TCGA)
Blood (GSE40279)
ChromHMM
LNCaP
Quiescent
Enhancer
Weak transcription
PrEC
Weak transcription
Enhancer
Weak transcription
Enhancer
Active TSS
Flanking active TSS
Active TSS
Bivalent Enhancer
Weak repressed polycomb
Enhancer
Weak transcription
Enhancer
Weak transcription

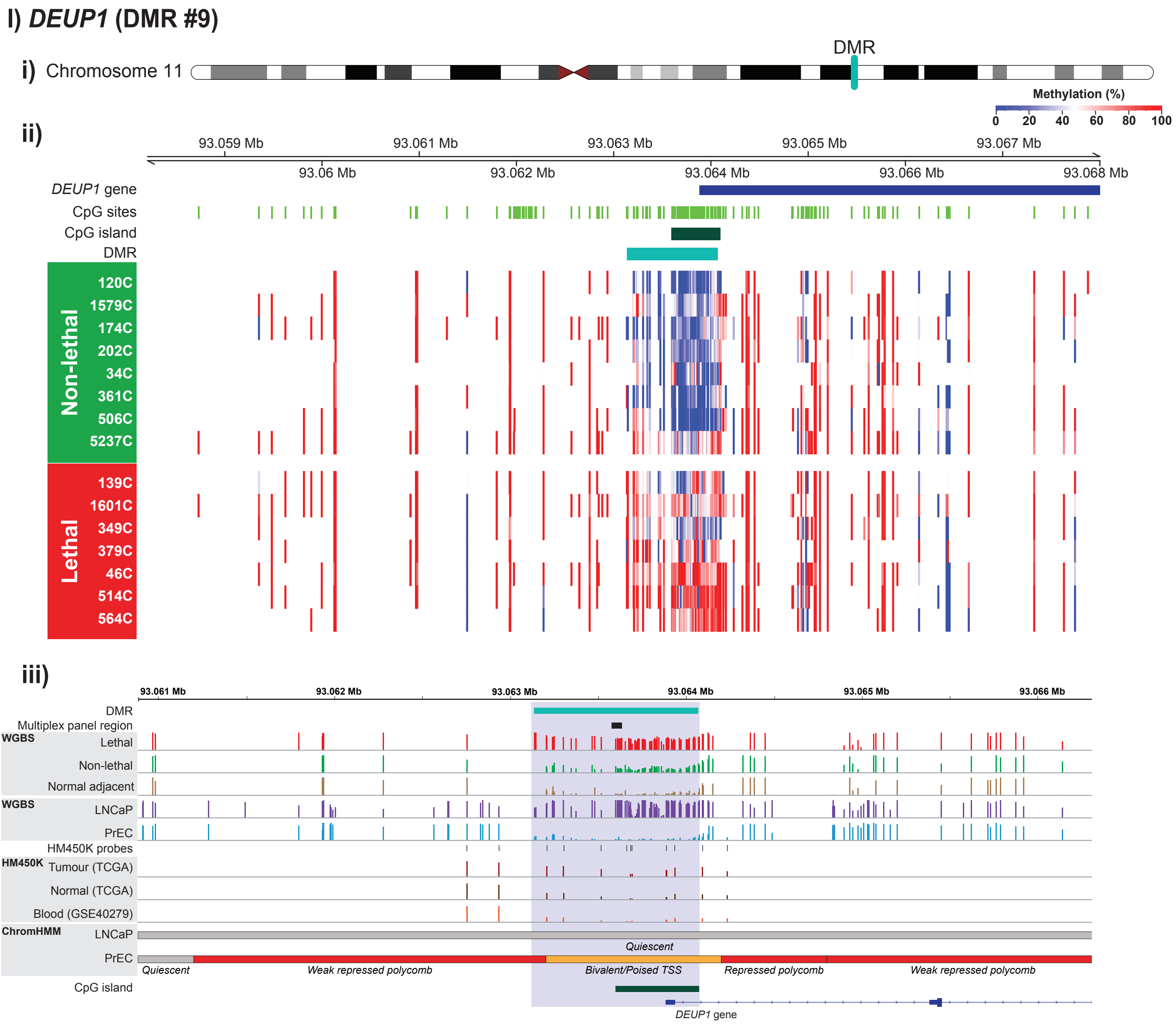

I) DEUP1 (DMR #9)
i) Chromosome 11
DMR
Methylation (%)
0 20 40 60 80 100
ii)
93.059 Mb
93.061 Mb
93.063 Mb
93.065 Mb
93.067 Mb
93.06 Mb
93.062 Mb
93.064 Mb
93.066 Mb
93.068 Mb
DEUP1 gene
CpG sites
CpG island
DMR
Non-lethal
120C
1579C
174C
202C
34C
361C
506C
5237C
Lethal
139C
1601C
349C
379C
46C
514C
564C
iii)
93.061 Mb
93.062 Mb
93.063 Mb
93.064 Mb
93.065 Mb
93.066 Mb
DMR
Multiplex panel region
WGBS
Lethal
Non-lethal
Normal adjacent
WGBS
LNCaP
PrEC
HM450K probes
HM450K
Tumour (TCGA)
Normal (TCGA)
Blood (GSE40279)
ChromHMM
LNCaP
Quiescent
PrEC
Quiescent
Weak repressed polycomb
Bivalent/Poised TSS
Repressed polycomb
Weak repressed polycomb
CpG island
DEUP1 gene

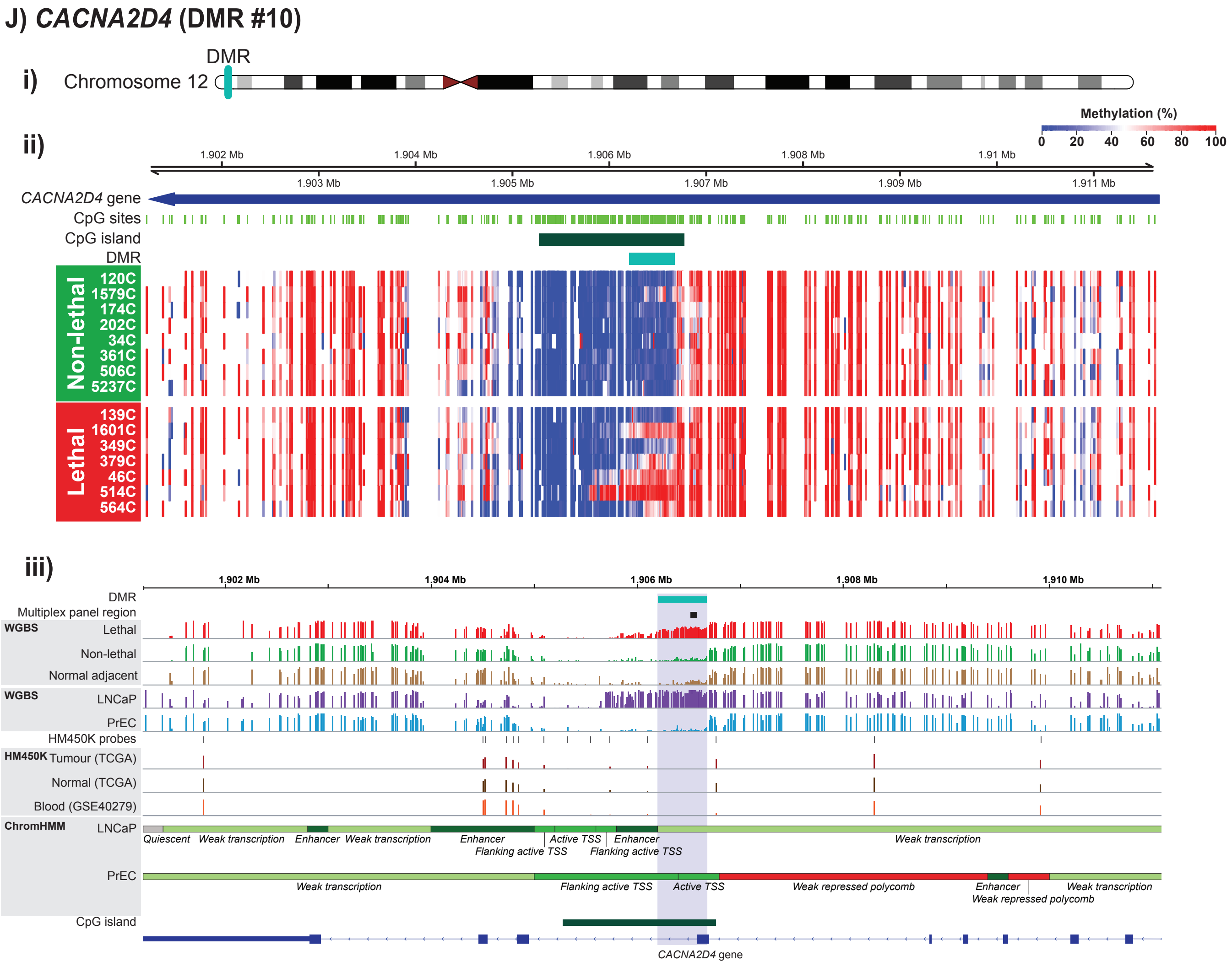
J) CACNA2D4 (DMR #10)
i)
DMR
Chromosome 12
ii)
Methylation (%)
0 20 40 60 80 100
1.902 Mb 1.904 Mb 1.906 Mb 1.908 Mb 1.91 Mb
1.903 Mb 1.905 Mb 1.907 Mb 1.909 Mb 1.911 Mb
CACNA2D4 gene
CpG sites
CpG island
DMR
Non-lethal
120C
1579C
174C
202C
34C
361C
506C
5237C
Lethal
139C
1601C
349C
379C
46C
514C
564C
iii)
1.902 Mb 1.904 Mb 1.906 Mb 1.908 Mb 1.910 Mb
DMR
Multiplex panel region
WGBS
Lethal
Non-lethal
Normal adjacent
WGBS
LNCaP
PrEC
HM450K probes
HM450K
Tumour (TCGA)
Normal (TCGA)
Blood (GSE40279)
ChromHMM
LNCaP
Quiescent Weak transcription Enhancer Weak transcription Enhancer Active TSS Enhancer Weak transcription
Flanking active TSS Flanking active TSS
PrEC
Weak transcription Flanking active TSS Active TSS Weak repressed polycomb Enhancer Weak transcription
Weak repressed polycomb
CpG island
CACNA2D4 gene

K) *CRACR2A* (DMR #11)

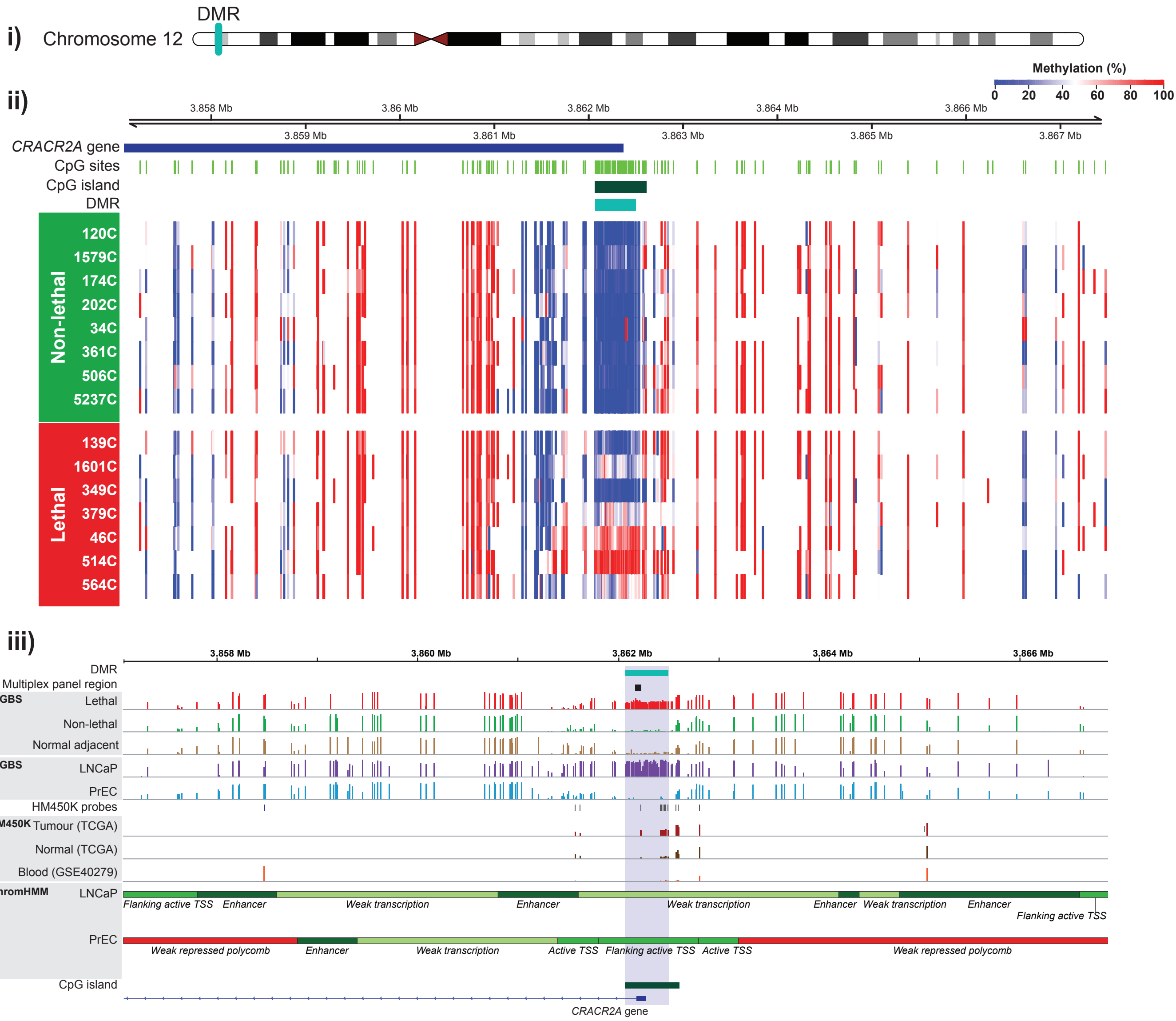

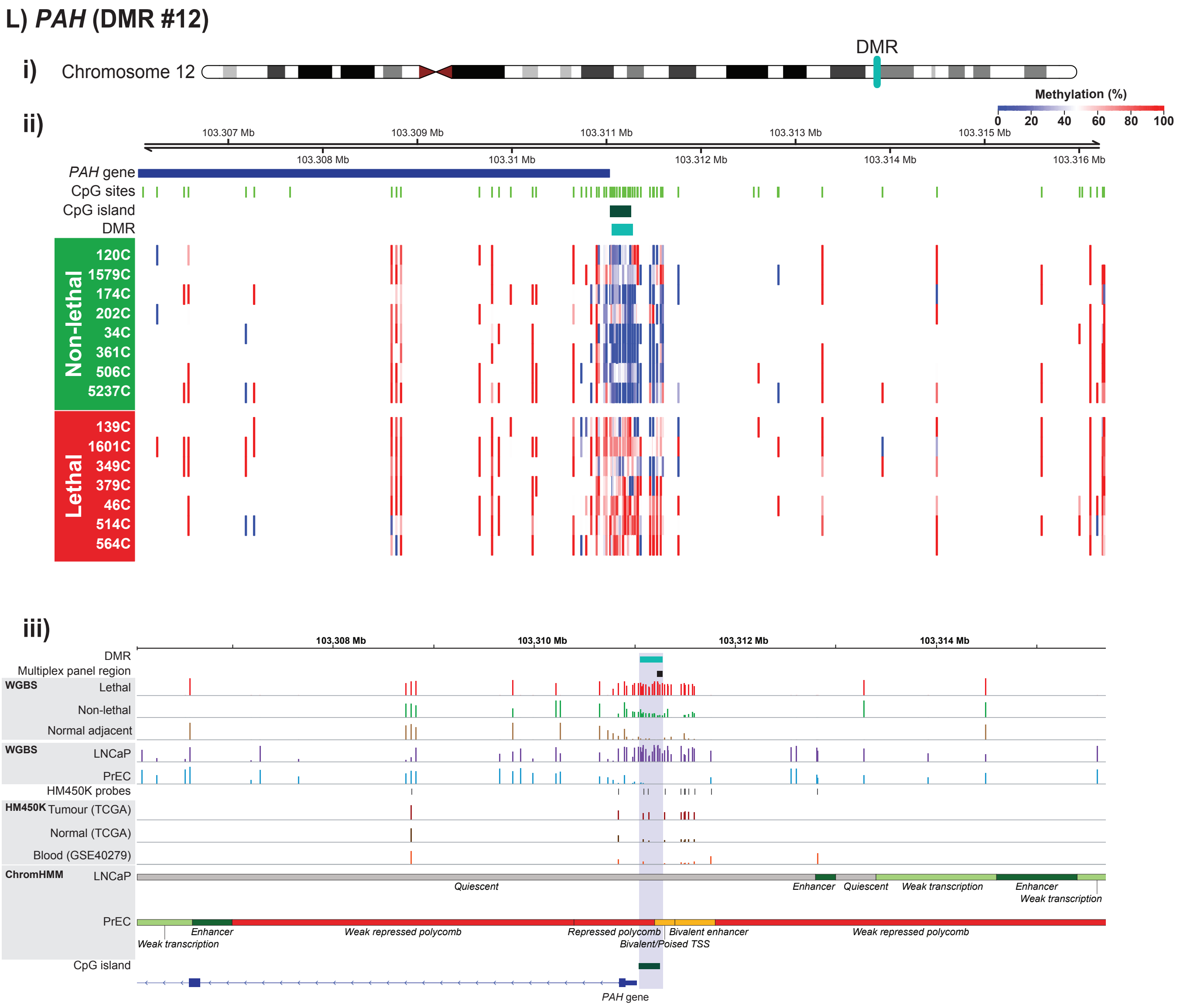

L) PAH (DMR #12)
i)
Chromosome 12
DMR
ii)
Methylation (%)
0 20 40 60 80 100
103.307 Mb
103.309 Mb
103.311 Mb
103.313 Mb
103.315 Mb
103.308 Mb
103.31 Mb
103.312 Mb
103.314 Mb
103.316 Mb
PAH gene
CpG sites
CpG island
DMR
Non-lethal
120C
1579C
174C
202C
34C
361C
506C
5237C
Lethal
139C
1601C
349C
379C
46C
514C
564C
iii)
103,308 Mb
103,310 Mb
103,312 Mb
103,314 Mb
DMR
Multiplex panel region
WGBS
Lethal
Non-lethal
Normal adjacent
WGBS
LNCaP
PrEC
HM450K probes
HM450K
Tumour (TCGA)
Normal (TCGA)
Blood (GSE40279)
ChromHMM
LNCaP
Quiescent
Enhancer
Quiescent
Weak transcription
Enhancer
Weak transcription
PrEC
Enhancer
Weak transcription
Weak repressed polycomb
Repressed polycomb
Bivalent/Poised TSS
Bivalent enhancer
Weak repressed polycomb
CpG island
PAH gene

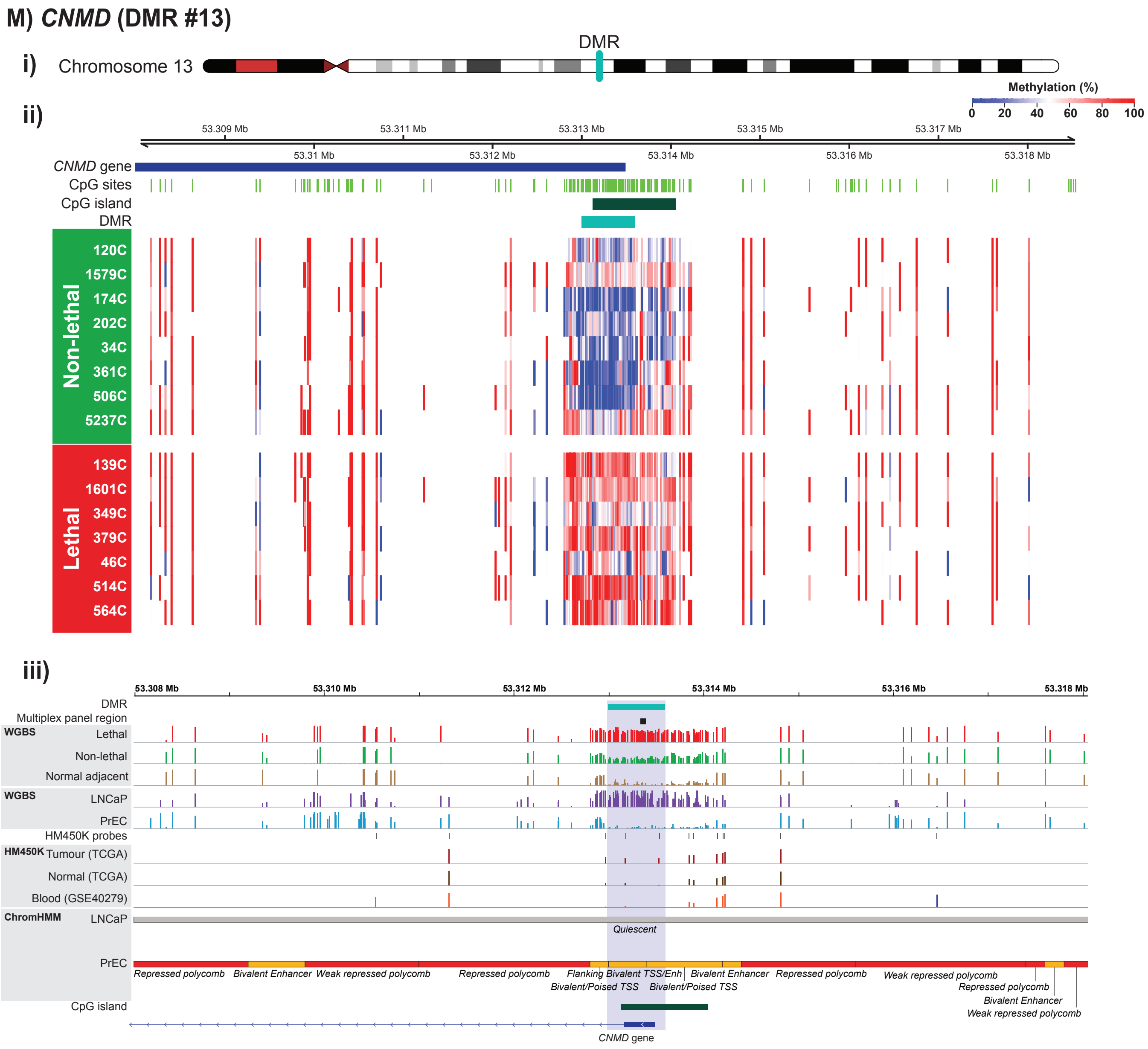

M) CNMD (DMR #13)
i)
Chromosome 13
DMR
ii)
Methylation (%)
0
20
40
60
80
100
53.309 Mb
53.311 Mb
53.313 Mb
53.315 Mb
53.317 Mb
53.31 Mb
53.312 Mb
53.314 Mb
53.316 Mb
53.318 Mb
CNMD gene
CpG sites
CpG island
DMR
Non-lethal
120C
1579C
174C
202C
34C
361C
506C
5237C
Lethal
139C
1601C
349C
379C
46C
514C
564C
iii)
53.308 Mb
53.310 Mb
53.312 Mb
53.314 Mb
53.316 Mb
53.318 Mb
DMR
Multiplex panel region
WGBS
Lethal
Non-lethal
Normal adjacent
WGBS
LNCaP
PrEC
HM450K probes
HM450K
Tumour (TCGA)
Normal (TCGA)
Blood (GSE40279)
ChromHMM
LNCaP
Quiescent
PrEC
Repressed polycomb
Bivalent Enhancer
Weak repressed polycomb
Repressed polycomb
Flanking Bivalent TSS/Enh
Bivalent Enhancer
Repressed polycomb
Weak repressed polycomb
Bivalent/Poised TSS
Bivalent/Poised TSS
Repressed polycomb
Bivalent Enhancer
Weak repressed polycomb
CpG island
CNMD gene

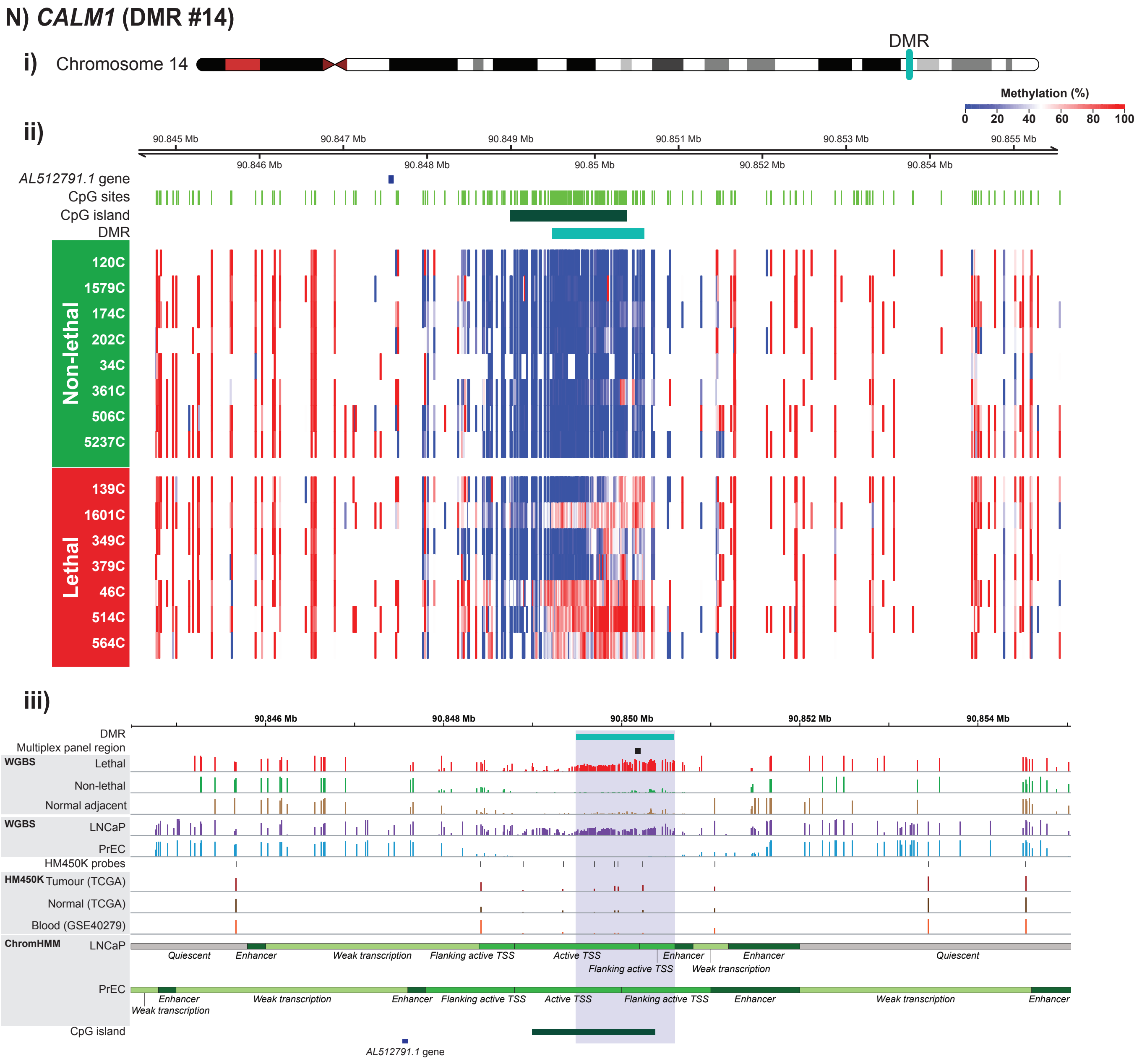
N) CALM1 (DMR #14)
i) Chromosome 14
DMR
Methylation (%)
0 20 40 60 80 100
ii)
90.845 Mb 90.847 Mb 90.849 Mb 90.851 Mb 90.853 Mb 90.855 Mb
90.846 Mb 90.848 Mb 90.85 Mb 90.852 Mb 90.854 Mb
AL512791.1 gene
CpG sites
CpG island
DMR
Non-lethal
120C
1579C
174C
202C
34C
361C
506C
5237C
Lethal
139C
1601C
349C
379C
46C
514C
564C
iii)
90.846 Mb 90.848 Mb 90.850 Mb 90.852 Mb 90.854 Mb
DMR
Multiplex panel region
WGBS Lethal
Non-lethal
Normal adjacent
WGBS LNCaP
PrEC
HM450K probes
HM450K Tumour (TCGA)
Normal (TCGA)
Blood (GSE40279)
ChromHMM LNCaP
Quiescent
Enhancer
Weak transcription
Flanking active TSS
Active TSS
Flanking active TSS
Enhancer
Weak transcription
Enhancer
Quiescent
PrEC
Enhancer
Weak transcription
Weak transcription
Enhancer
Flanking active TSS
Active TSS
Flanking active TSS
Enhancer
Weak transcription
Enhancer
CpG island
AL512791.1 gene

O) *PARP6* (DMR #15)

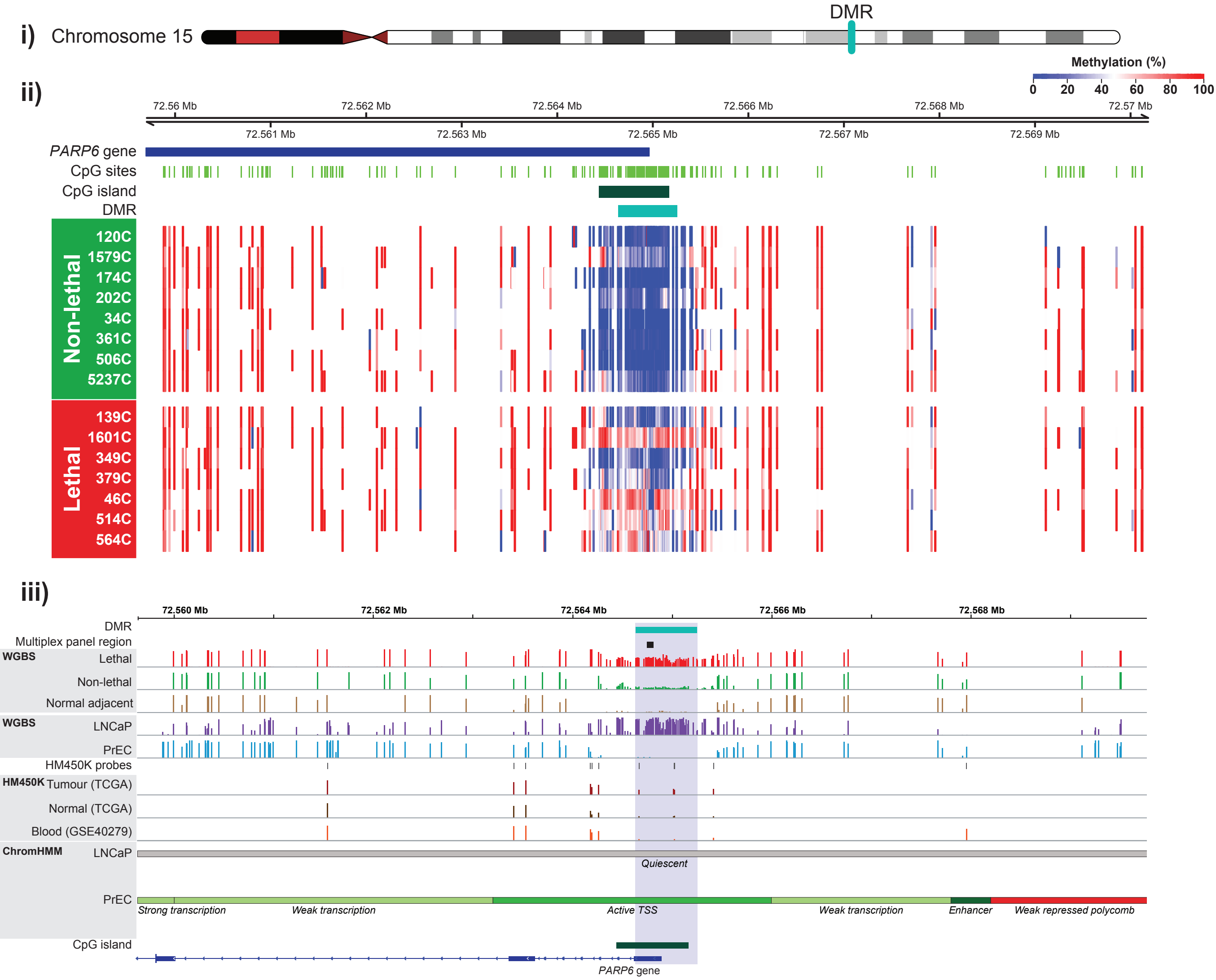

P) *LRRC37A3* (DMR #16)

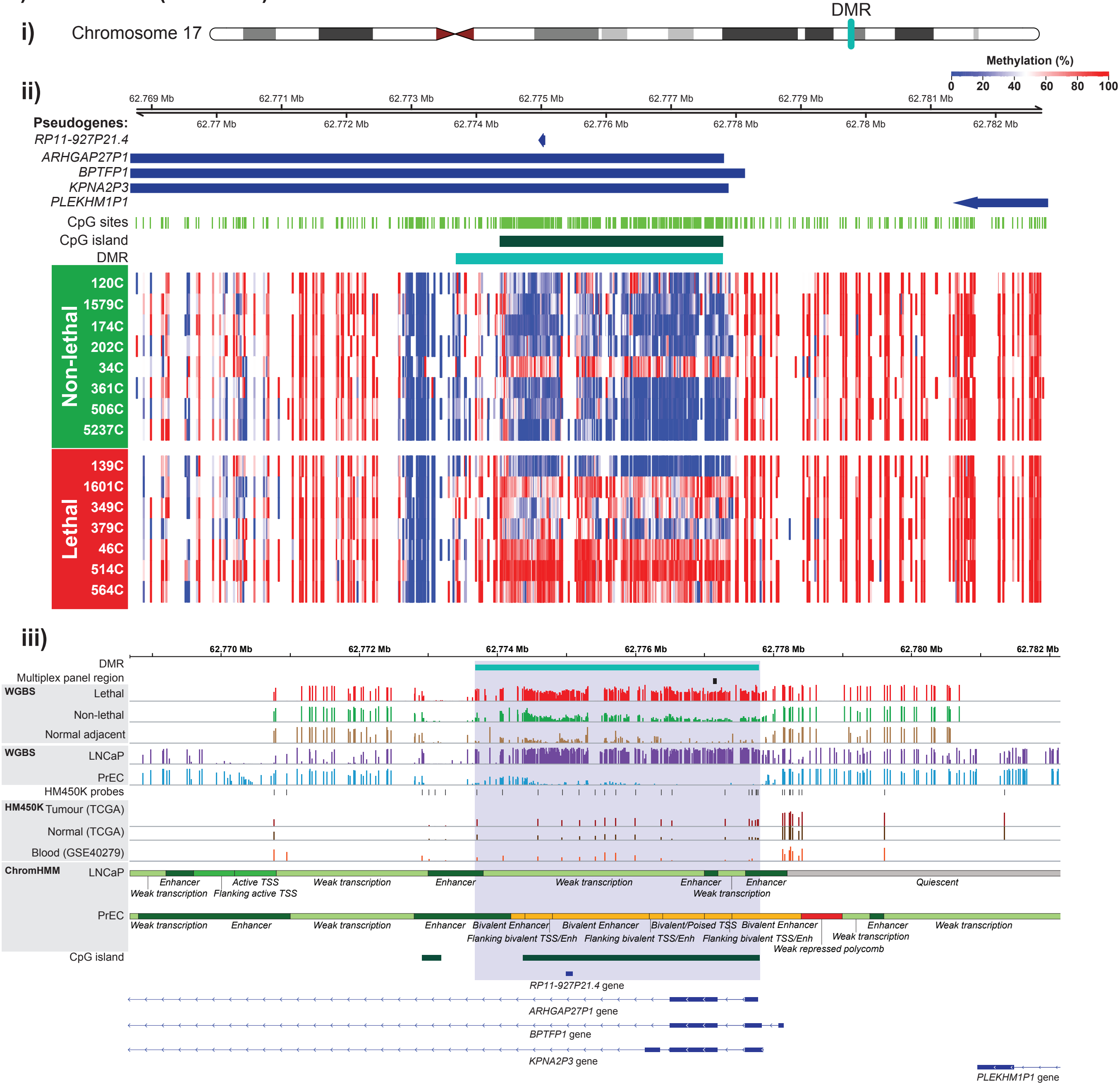

Q) *TBX1* (DMR #17)

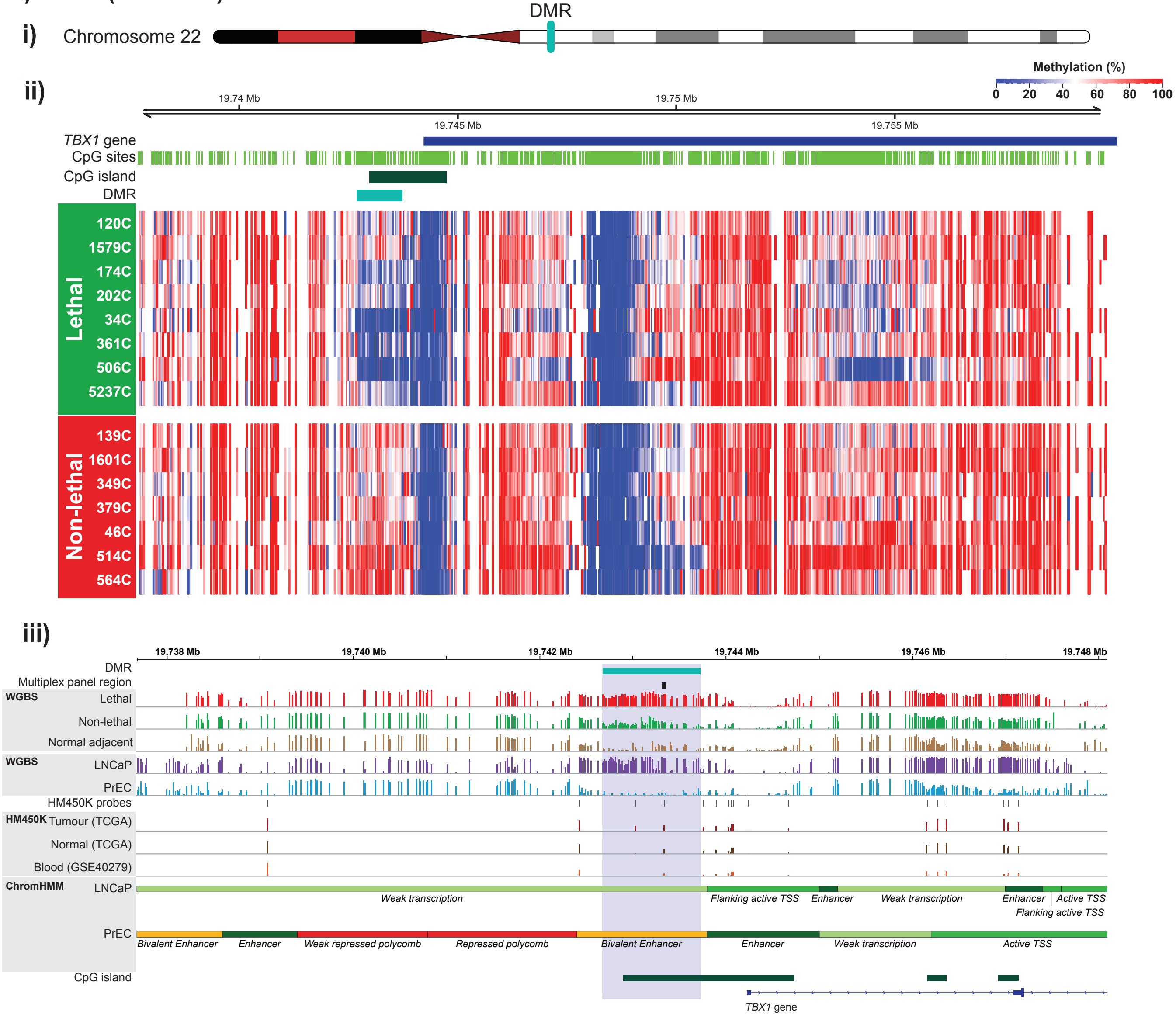

i)
Chromosome 22
DMR
ii)
Methylation (%)
0
20
40
60
80
100
19.74 Mb
19.75 Mb
19.745 Mb
19.755 Mb
TBX1 gene
CpG sites
CpG island
DMR
Lethal
120C
1579C
174C
202C
34C
361C
506C
5237C
Non-lethal
139C
1601C
349C
379C
46C
514C
564C
iii)
19.738 Mb
19.740 Mb
19.742 Mb
19.744 Mb
19.746 Mb
19.748 Mb
DMR
Multiplex panel region
WGBS
Lethal
Non-lethal
Normal adjacent
WGBS
LNCaP
PrEC
HM450K probes
HM450K
Tumour (TCGA)
Normal (TCGA)
Blood (GSE40279)
ChromHMM
LNCaP
Weak transcription
Flanking active TSS
Enhancer
Weak transcription
Enhancer
Active TSS
Flanking active TSS
PrEC
Bivalent Enhancer
Enhancer
Weak repressed polycomb
Repressed polycomb
Bivalent Enhancer
Enhancer
Weak transcription
Active TSS
CpG island
TBX1 gene

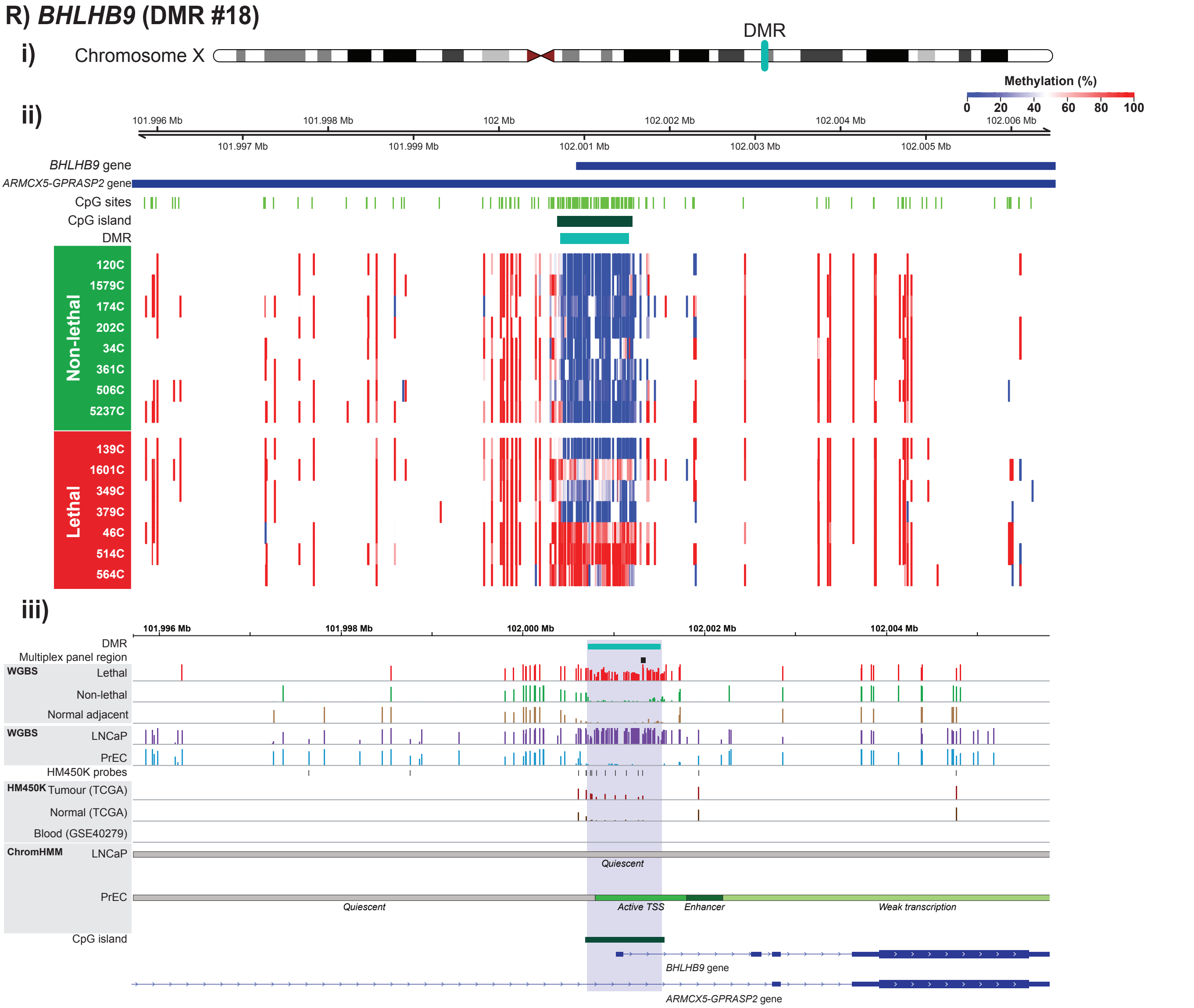

Figure S1. Data used to identify and prioritise 18 DMRs (A-R) for biomarker panel: (i) Ideogram showing location of DMR (turquoise bar) within chromosome (ii) DMRCate heatmap of the DMR (turquoise bar) region showing methylation across individual patients in the non-lethal (green) vs lethal (red) groups. The genes (if within the region shown) are represented by a dark blue bar, with CpG islands represented by dark green bars. (iii) IGV plot of the DMR (turquoise bar) region showing average methylation across patients in the lethal group (red bars) vs non-lethal group (green bars) and normal adjacent prostate tissue (dark brown bars), methylation in LNCaP (purple bars) vs PrEC (blue bars) cell lines. The HM450K array probes are shown in light grey, with methylation in tumour samples (TCGA) shown in dark red, normal samples (TCGA) in dark brown, and blood methylation (GSE40279) in orange. The region targeted in the multiplex panel is represented by a black bar, CpG island represented by the dark green bar, with ChromHMM states for LNCaP and PrEC shown across the DMR. The putative regulatory elements from ChromHMM segmentation data have been grouped into Promoter (Active TSS, Flanking active TSS) (green), Transcription (Transcription at 5' and 3', Strong transcription, Weak transcription) (light green), Enhancer (Genic enhancer, Enhancer) (dark green), Bivalent (Bivalent/Poised TSS, Flanking bivalent TSS, Bivalent Enhancer) (light orange), Repressive (ZNF genes and repeats, Heterochromatin, Repressed Polycomb, Weak repressed polycomb) (red) and Quiescent (light grey).

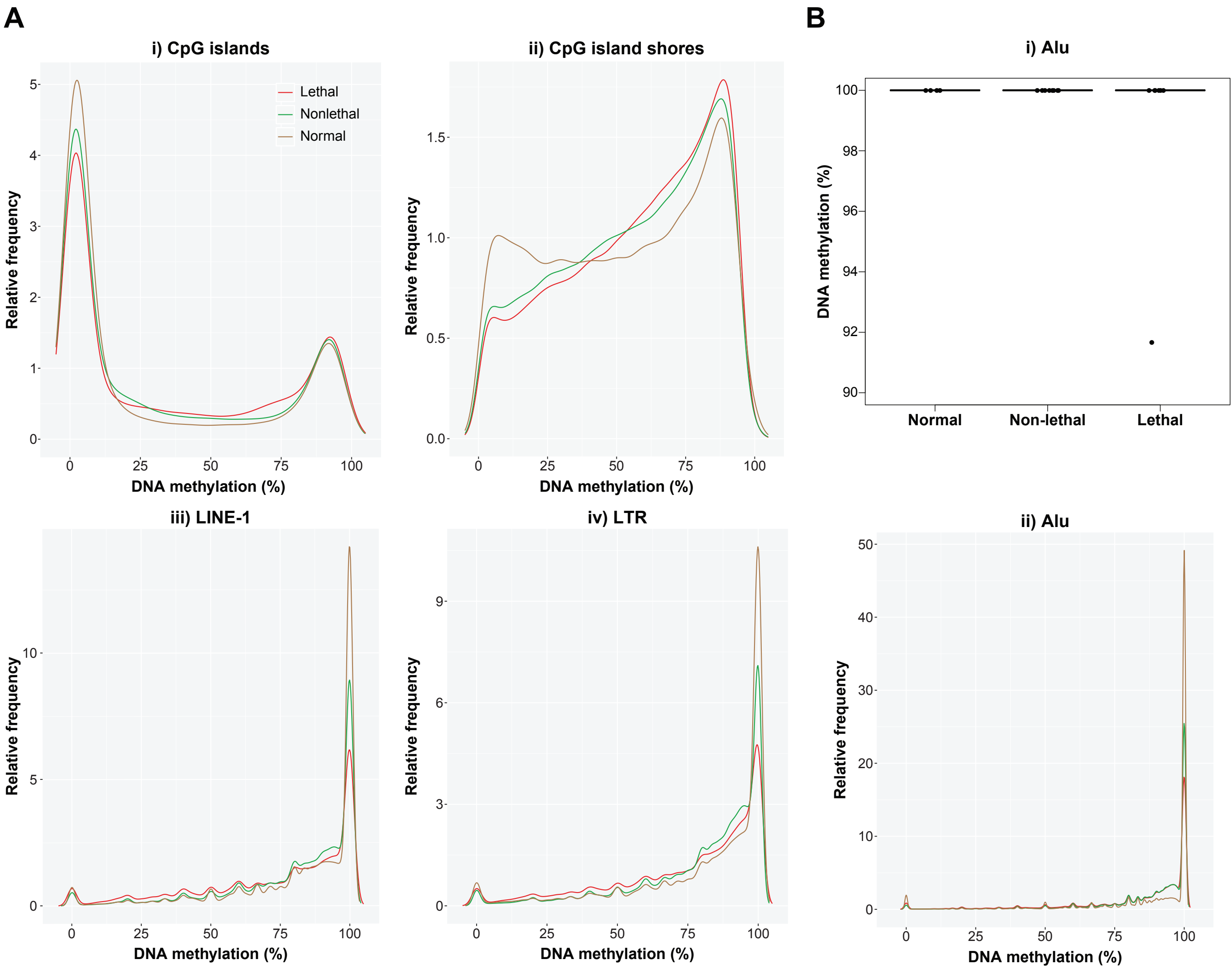

Figure S2. Distribution of DNA methylation levels in different genomic contexts. (A) Plots of the distribution of genome-wide DNA methylation levels in normal adjacent tissue (light brown), and cancer tissue non-lethal (green) and lethal (red) patients in: i) CpG islands, ii) CpG island shores, iii) LINE-1 repetitive elements and iv) Long tandem repeats (LTRs). (B) i) Boxplots and ii) distribution plot of DNA methylation levels across Alu repetitive elements in normal adjacent tissue, and cancer tissue from non-lethal and lethal patients.

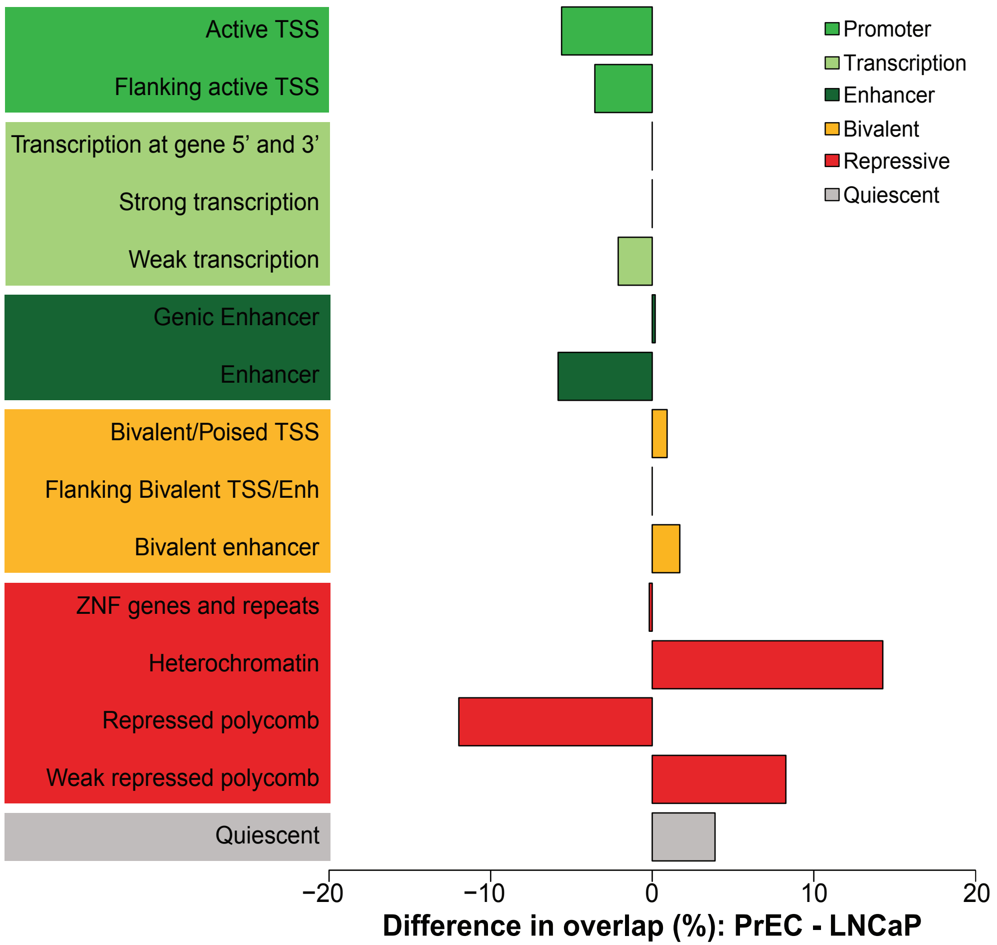

Figure S3. Bargraph of the percentage difference in overlap between PrEC and LNCaP ChromHMM states with all hypomethylated DMRs. The putative regulatory elements from ChromHMM segmentation data have been grouped into Promoter (Active TSS, Flanking active TSS) (green), Transcription (Transcription at 5' and 3', Strong transcription, Weak transcription) (light green), Enhancer (Genic enhancer, Enhancer) (dark green), Bivalent (Bivalent/Poised TSS, Flanking bivalent TSS, Bivalent Enhancer) (light orange), Repressive (ZNF genes and repeats, Heterochromatin, Repressed Polycomb, Weak repressed polycomb) (red) and Quiescent (light grey).

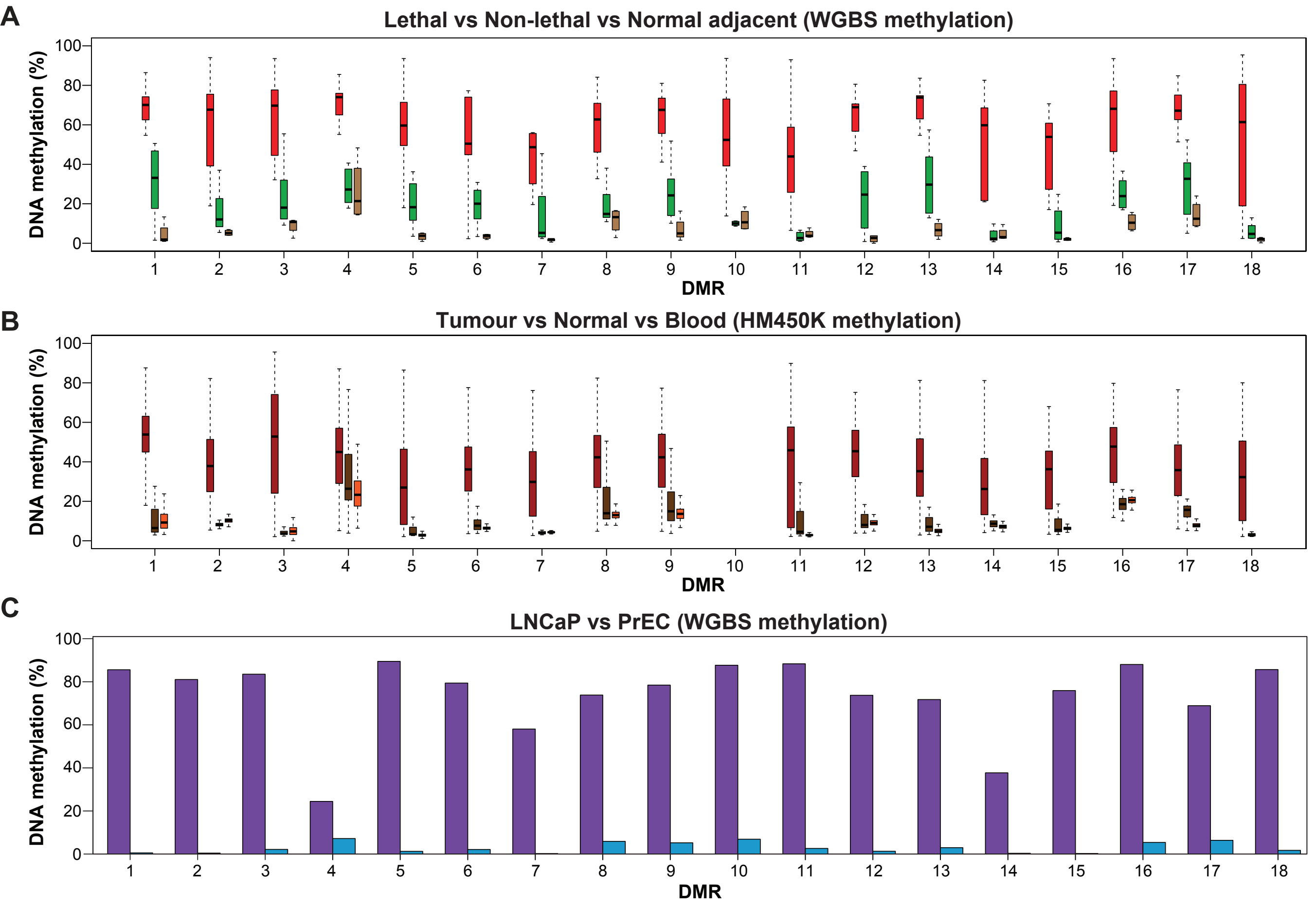

Figure S4. Selection of DMRs for the biomarker panel. (A) Boxplots of WGBS methylation in lethal (red), non-lethal (green), and normal adjacent (light brown) patient samples across the 18 selected DMRs. (B) Boxplots of methylation in tumour (dark red) and normal (dark brown) samples (TCGA) and blood samples (GEO GSE40279) across the 18 selected DMRs. (C) Bargraphs of methylation in LNCaP (purple) and PrEC (blue) cell lines across the 18 selected DMRs.

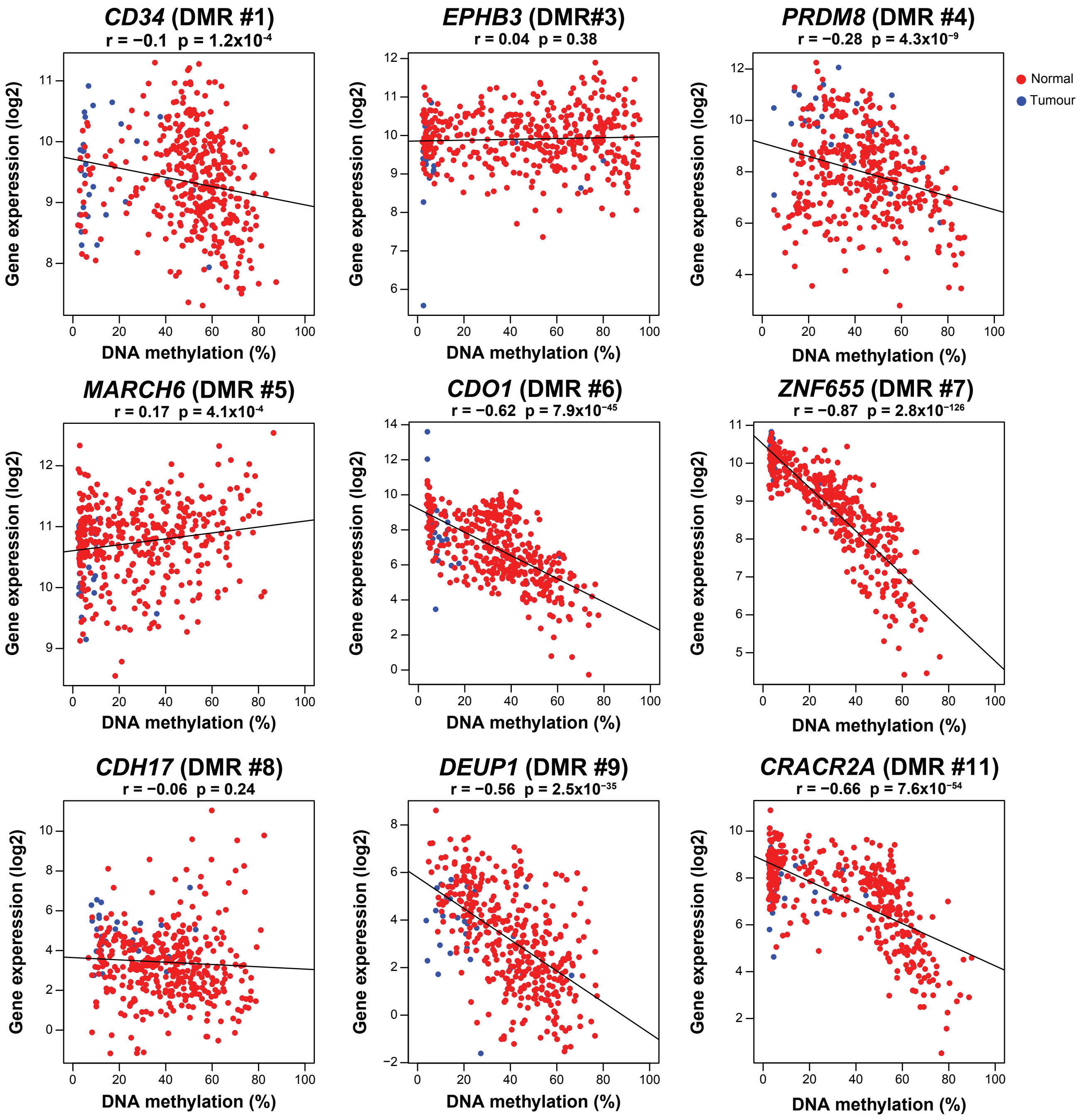
CD34 (DMR #1)
r = −0.1 p = 1.2x10−4
EPHB3 (DMR#3)
r = 0.04 p = 0.38
PRDM8 (DMR #4)
r = −0.28 p = 4.3x10−9
Normal
Tumour
MARCH6 (DMR #5)
r = 0.17 p = 4.1x10−4
CDO1 (DMR #6)
r = −0.62 p = 7.9x10−45
ZNF655 (DMR #7)
r = −0.87 p = 2.8x10−126
CDH17 (DMR #8)
r = −0.06 p = 0.24
DEUP1 (DMR #9)
r = −0.56 p = 2.5x10−35
CRACR2A (DMR #11)
r = −0.66 p = 7.6x10−54
Gene experession (log2)
DNA methylation (%)

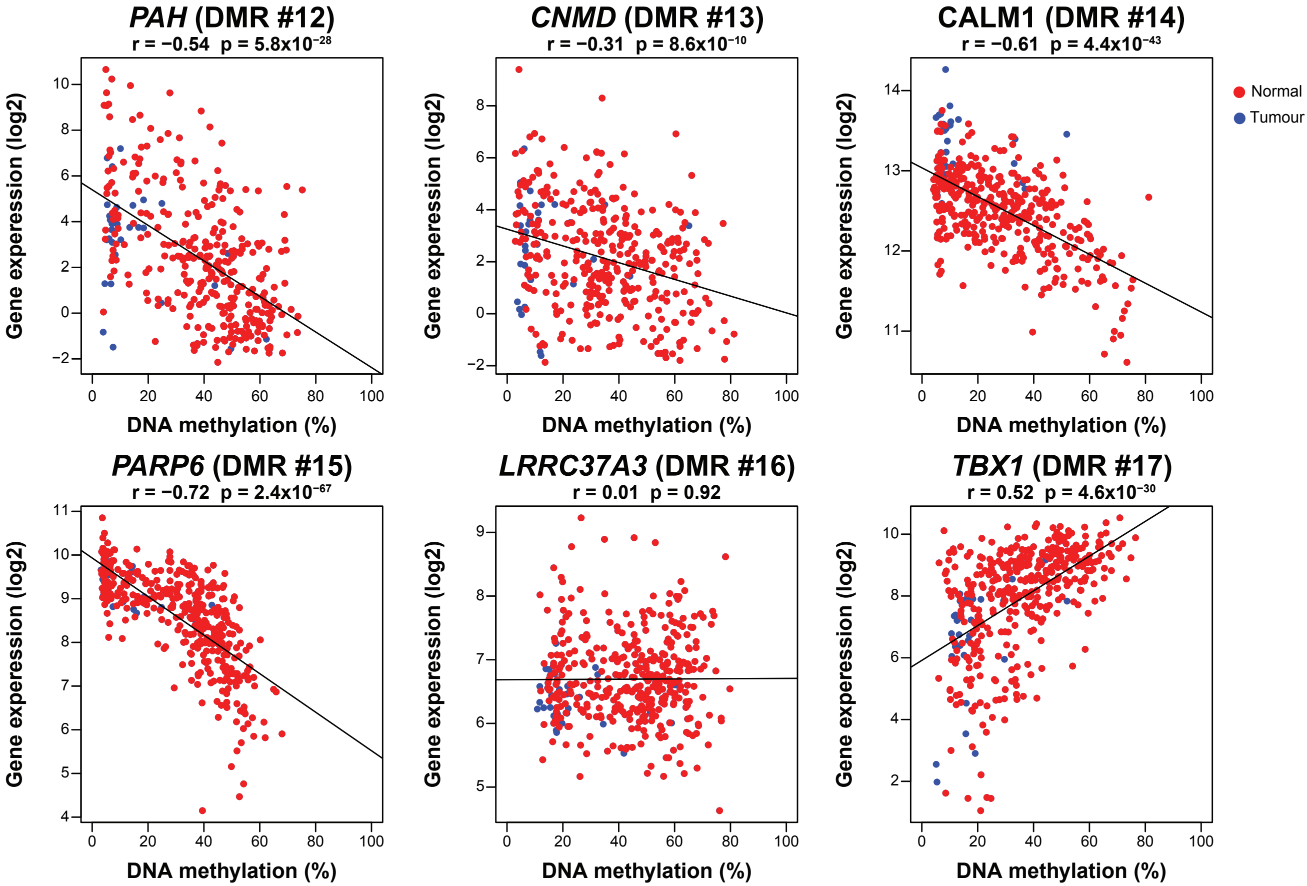

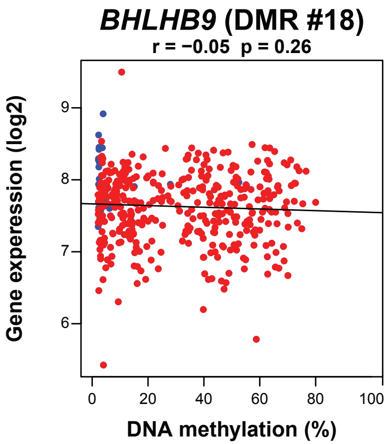

Figure S5. Pearson's correlation between DMR methylation and expression in TCGA. Scatterplot of mean methylation of HM450 probes across DMR (%) and RNA-seq gene expression(log2) of the nearest-proteincoding gene in TCGA normal (blue) and tumour (red) tissue.

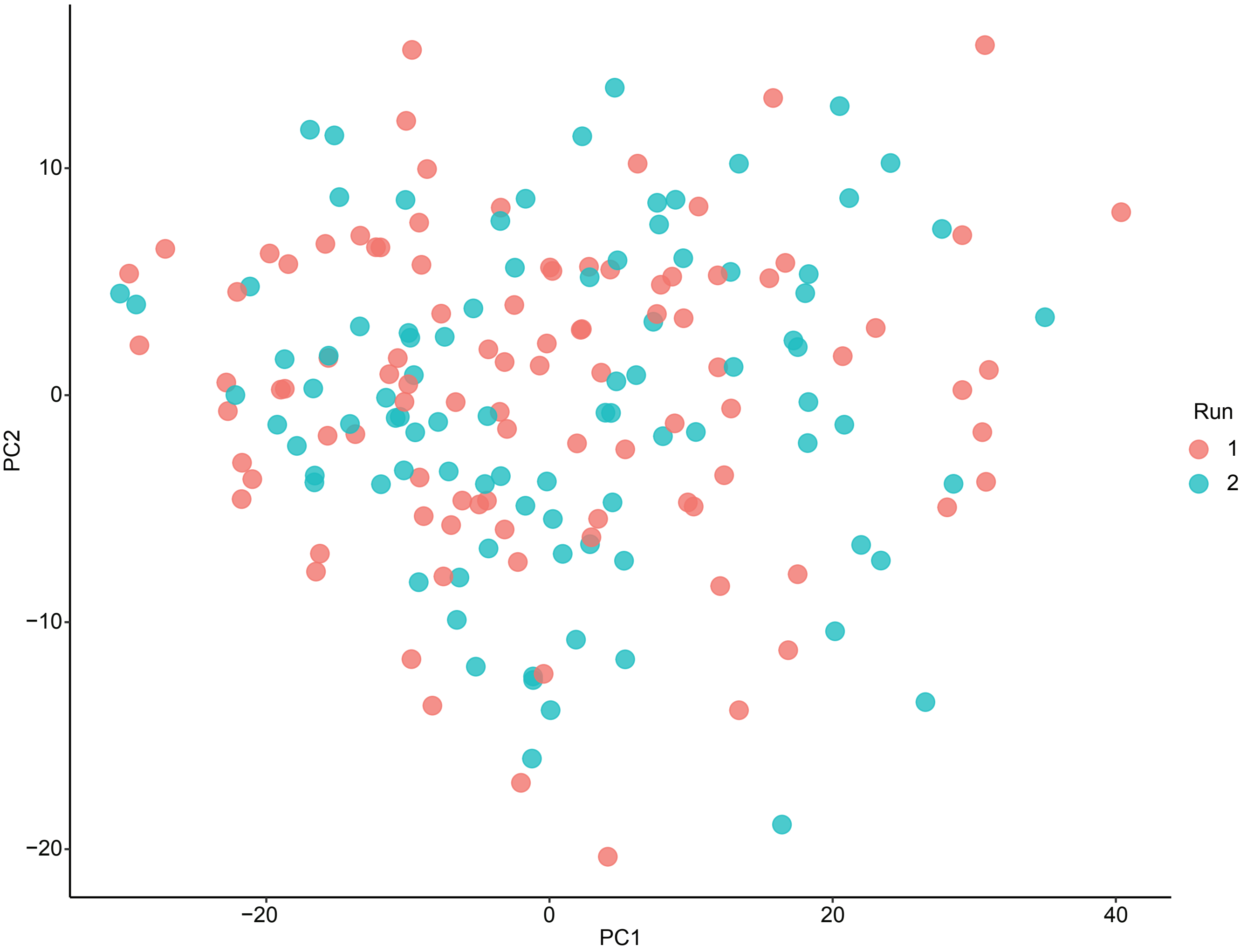

Figure S6. Batch effects. MDS plot of MBPS methylation data showing no evidence of batch effects between the different sequencing runs. Red = sequencing run 1; blue = sequencing run 2.

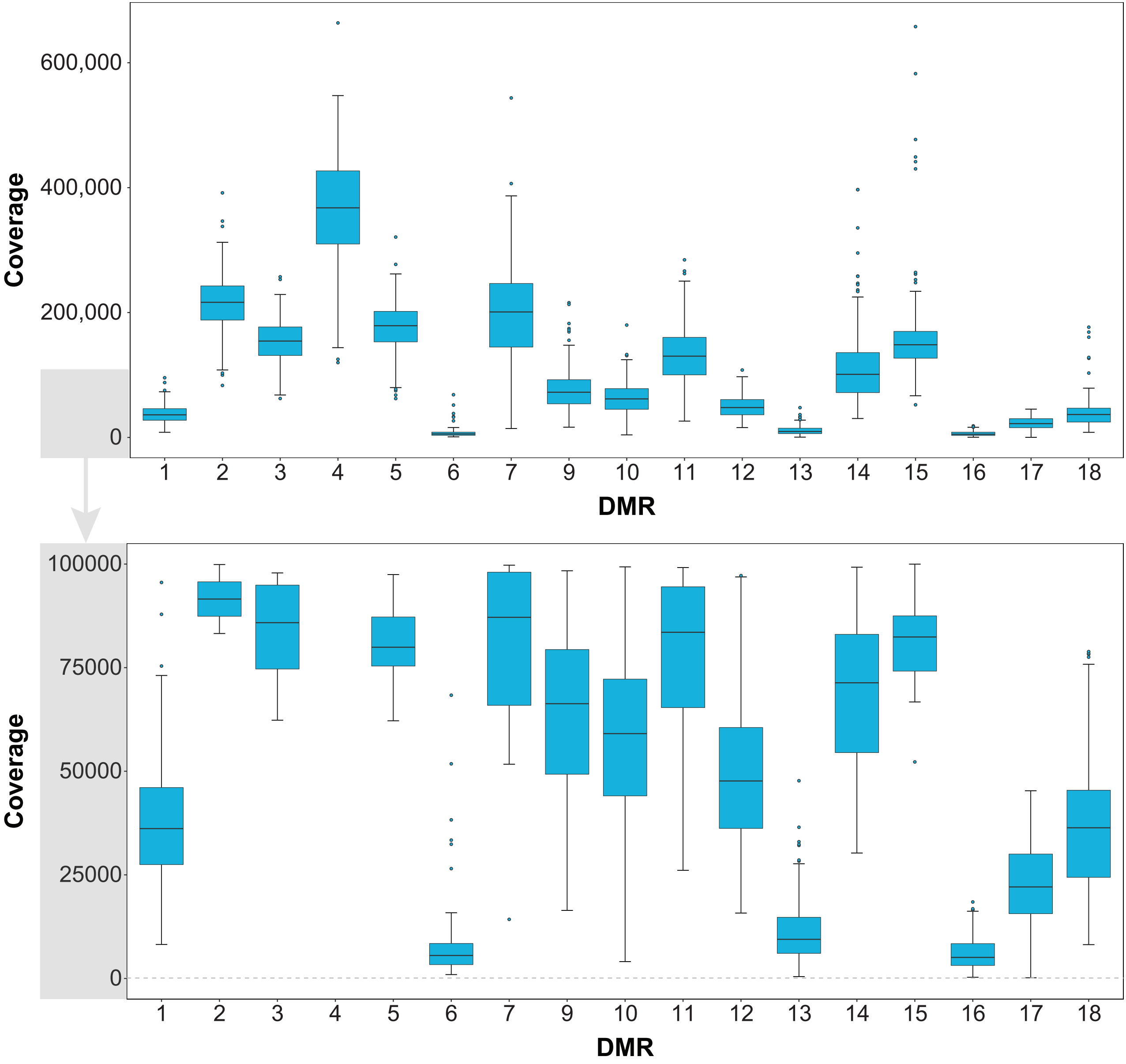

Figure S7. Sequencing coverage. Boxplot showing full coverage (top panel) across the 17 genomic regions passing quality control in the targeted MBPS panel from a sequencing run on n = 186 RP samples. Bottom panel shows the same data with a different y-axis scale to better show the difference between lower coverage amplicons, with the dashed line indicating the cut-off (100 reads).

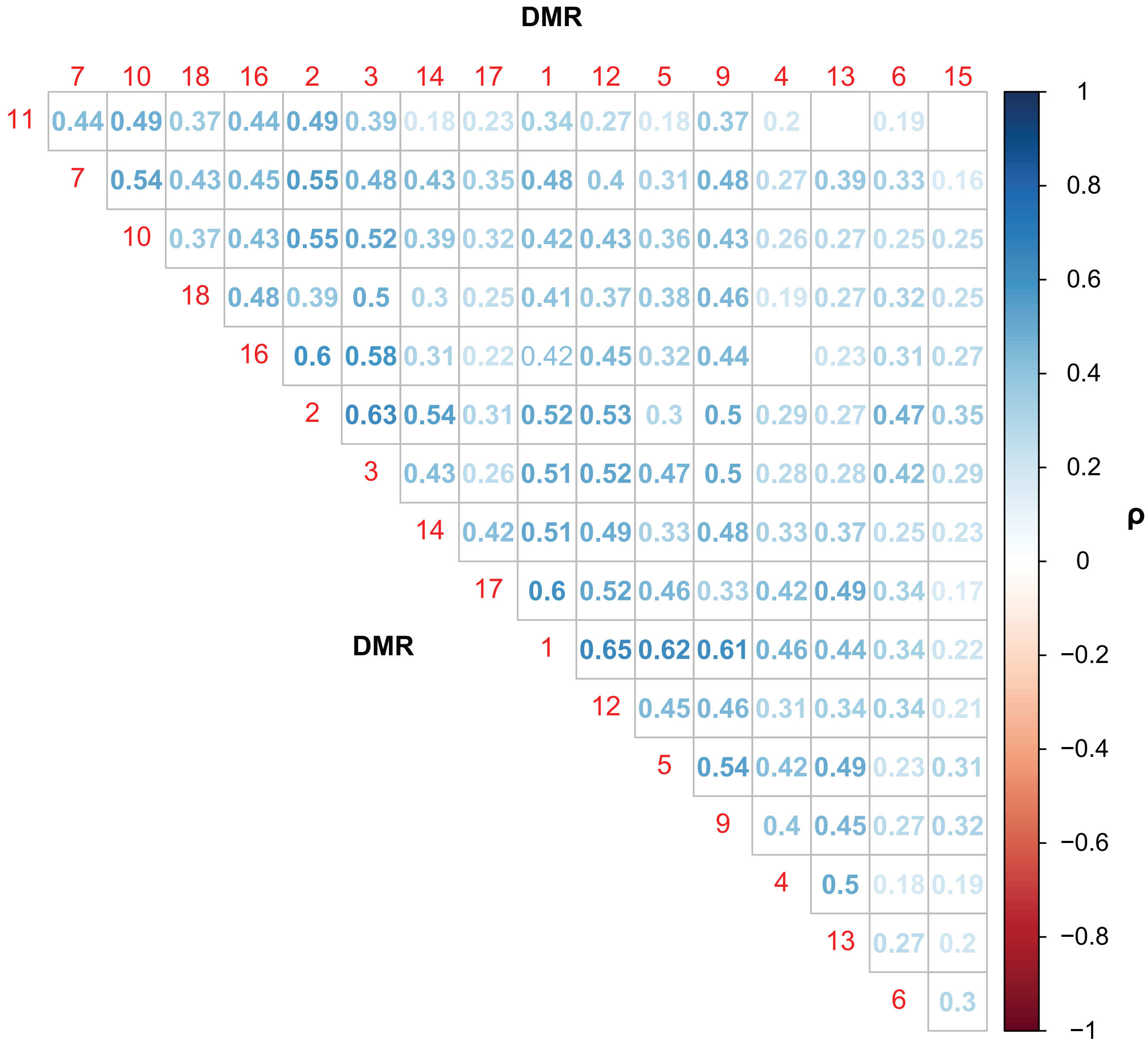

Figure S8. Pairwise correlations between methylation levels at the 17 genomic regions. Spearman's rank correlation coefficient (ρ) is presented in the boxes, with blank squares indicating p > 0.05 (not significant).

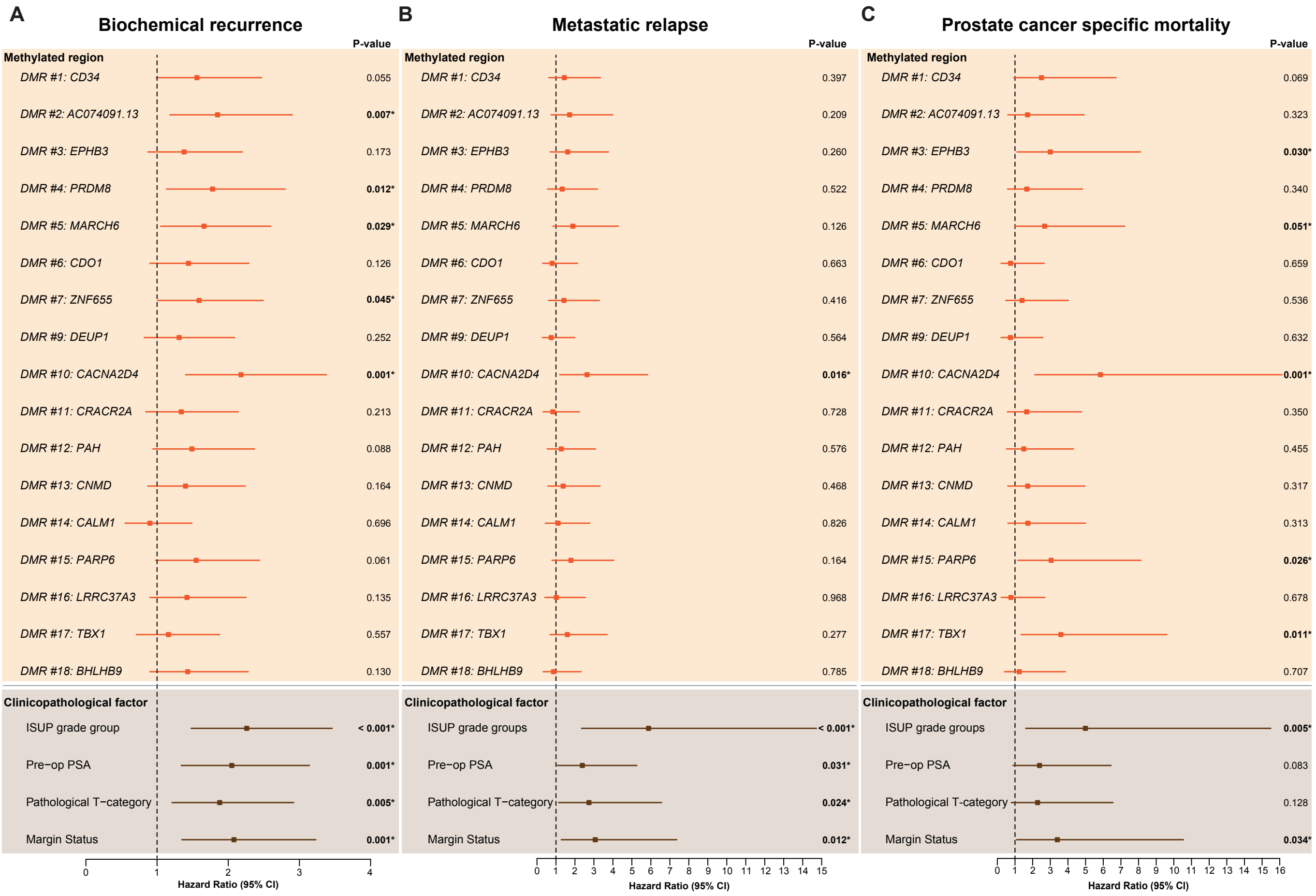

Figure S9. Univariable Cox regression analysis. Forest plots showing the hazard ratio and 95% CI for all 17 methylated genomic regions and 4 clinicopathological markers in univariable Cox regression analysis for (A) Biochemical recurrence, (B) Metastatic relapse and (C) Prostate cancer specific mortality. Methylated regions are shaded in orange, and clinicopathological factors are shaded in brown.

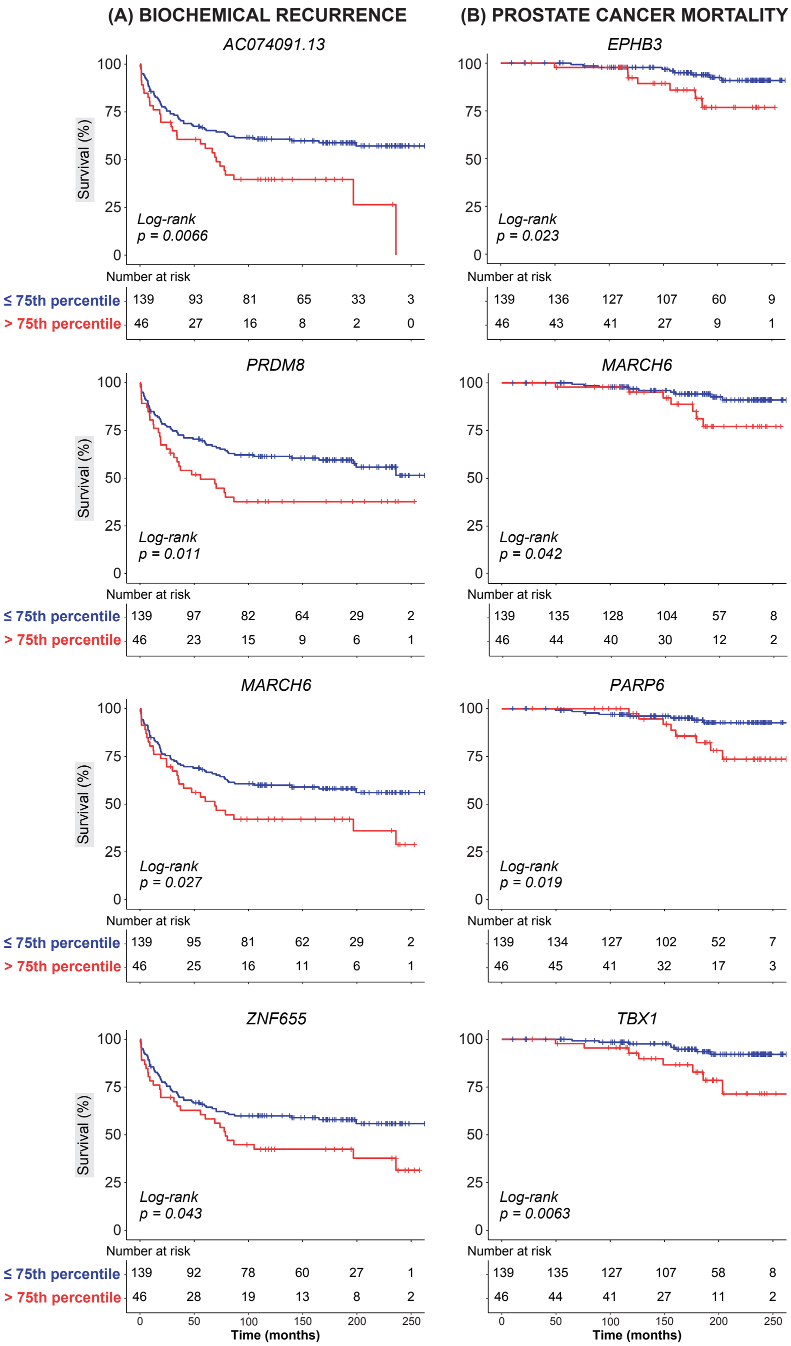

Figure S10. Kaplan-Meier survival curves: Methylation. Kaplan-Meier survival curves of methylation at genomic regions with significant associations with (A) Biochemical Recurrence, (B) Prostate cancer specific mortality in log-rank analysis. Red line indicates higher methylation (> 75th percentile), blue line indicates lower methylation (≤ 75th percentile).

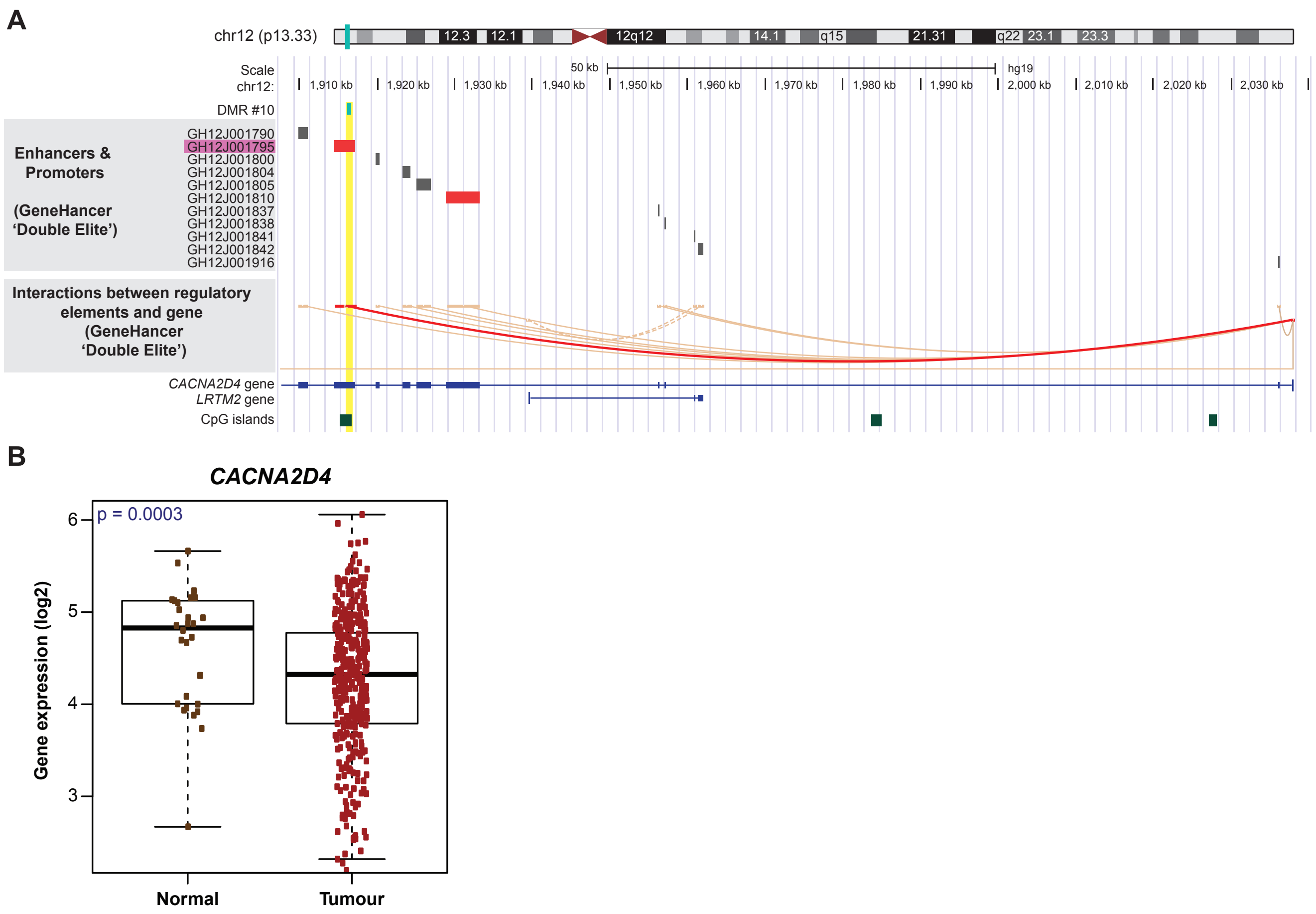

Figure S11. Characterisation of the potential regulatory role of the *CACNA2D4* DMR. (A) UCSC genome browser image of the *CACNA2D4* DMR (DMR #10) (turquoise bar, highlighted in yellow). The Genehancer Double Elite enhancer (grey boxes) and promoter (light red boxes) regulatory regions are shown, with the regulatory element (GH12J001795) overlapping the DMR highlighted in pink. Interactions between Genehancer regulatory elements and target genes are shown in cream coloured loops, with the loop connecting the DMR #10 and the *CACNA2D4* TSS shown in red. The two target genes within this region, *CACNA2D4* and *LRTM2* are shown in dark blue, with CpG islands in dark green. (B) Boxplot of *CACNA2D4* gene expression (log2) in TCGA normal (dark brown) and tumour (dark red) tissue, with p-value from t-test shown.
